# Comparative Transcriptome Analyses of *Schistosoma japonicum* Derived From SCID Mice and BALB/c Mice: Clues to the Abnormality in Parasite Growth and Development

**DOI:** 10.3389/fmicb.2020.00274

**Published:** 2020-03-11

**Authors:** Rong Liu, Wen-Jun Cheng, Feng Ye, Yao-Dan Zhang, Qin-Ping Zhong, Hui-Fen Dong, Hong-Bin Tang, Hong Jiang

**Affiliations:** ^1^School of Basic Medical Sciences, Wuhan University, Wuhan, China; ^2^Laboratory Animal Center, School of Medicine, Wuhan University, Wuhan, China

**Keywords:** SCID mouse, BALB/c mouse, *Schistosoma japonicum*, growth and development, transcriptome sequencing, dsRNA, RNA interference *in vivo*

## Abstract

Schistosomiasis, caused by the parasitic flatworms called schistosomes, remains one of the most prevailing parasitic diseases in the world. The prodigious oviposition of female worms after maturity is the main driver of pathology due to infection, yet our understanding about the regulation of development and reproduction of schistosomes is limited. Here, we comparatively profiled the transcriptome of *Schistosoma japonicum* recovered from SCID and BALB/c mice, which were collected 35 days post-infection, when prominent morphological abnormalities could be observed in schistosomes from SCID mice, by performing RNA-seq analysis. Of the 11,183 identified genes, 62 differentially expressed genes (DEGs) with 39 upregulated and 23 downregulated messenger RNAs (mRNAs) were found in male worms from SCID mice (S_M) *vs*. male worms from BALB/c mice (B_M), and 240 DEGs with 152 upregulated and 88 downregulated mRNAs were found in female worms from SCID mice (S_F) *vs*. female worms from BALB/c mice (B_F). We also tested nine DEGs with a relatively higher expression abundance in the gonads of the worms (ovary, vitellaria, or testis), suggesting their potential biological significance in the development and reproduction of the parasites. Gene ontology (GO) enrichment analysis revealed that GO terms such as “microtubule-based process,” “multicellular organismal development,” and “Rho protein signal transduction” were significantly enriched in the DEGs in S_F *vs*. B_F, whereas GO terms such as “oxidation–reduction process,” “response to stress,” and “response to DNA damage stimulus” were significantly enriched in the DEGs in S_M *vs*. B_M. These results revealed that the differential expression of some important genes might contribute to the morphological abnormalities of worms in SCID mice. Furthermore, we selected one DEG, the mitochondrial prohibitin complex protein 1 (*Phb1*), to perform double-stranded RNA (dsRNA)-mediated RNA interference (RNAi) *in vivo* targeting the worms in BALB/c mice, and we found that it was essential for the growth and reproductive development of both male and female *S. japonicum* worms. Taken together, these results provided a wealth of information on the differential gene expression profiles of schistosomes from SCID mice when compared with those from BALB/c mice, which were potentially involved in regulating the growth and development of schistosomes. These findings contributed to an understanding of parasite biology and provided a rich resource for the exploitation of antischistosomal intervention targets.

## Introduction

Schistosomiasis, caused by infection with the blood-dwelling endoparasites of the genus *Schistosoma* (the only digenetic trematodes), remains one of the most serious parasitic diseases worldwide, afflicting more than 200 million people and generating annual losses of 1.7–4.5 million disability-adjusted life years (DALYs) of humans, with about 800 million at risk (King et al., 2005; Steinmann et al., 2006; Gray et al., 2010; Ross et al., 2013; WHO, 2016). Schistosomes have a complex life cycle involving both a snail intermediate and a vertebrate definitive host and have coevolved a long-lived and intricate relationship with their human hosts (Harrison and Doenhoff, 1983; Amiri et al., 1992; Nakazawa et al., 1997; Cheever et al., 1999; Hernandez et al., 2004; Blank et al., 2006; Liu et al., 2006; deWalick et al., 2012), as well as an indispensable interplay between the adult male and female parasites during their development and maturation (Den Hollander and Erasmus, 1985; Cai et al., 2016; Wang J. et al., 2017). As the only members of the trematodes, schistosomes have evolved to form separate sexes—dioecious (Platt and Brooks, 1997; Kunz, 2001). Furthermore, mating is required to induce sexual development and maturation of the female worms as a prerequisite for egg production (Kunz, 2001; Ross et al., 2002; Hu et al., 2004; Quack et al., 2006; Beckmann et al., 2010; Lu et al., 2016). The latter finally leads to inflammatory processes in the affected organs of their host (such as the gut and liver of the hosts infected with *Schistosoma japonicum* or *Schistosoma mansoni* parasites) due to egg deposition, culminating in the formation of granulomas and fibrosis (Ross et al., 2002). The clinical signs of schistosomiasis are dependent on the maturation stage of the parasites and their eggs. In humans, acute infection is characterized by a debilitating febrile illness (Katayama fever) that usually occurs before the appearance of eggs in the stool, with a peak at 6–8 weeks post-infection. In chronic diseases, eggs trapped in various tissues induce the formation of granulomatous inflammation, which subsequently results in fibrosis that causes many of the pathological conditions.

It is believed that parasites definitely cause damage to their host, and the host resists against the invading parasites by its immune response (Han et al., 2009; The Schistosoma japonicum Genome Sequencing and Functional Analysis Consortium, 2009; deWalick et al., 2012; Duan et al., 2014; Wang J. et al., 2014; Morais et al., 2018; Roderfeld et al., 2018). However, several studies have already observed that host factors such as the immune factors can modulate parasite development and maturation (Amiri et al., 1992; Hernandez et al., 2004; LoVerde et al., 2009; Beckmann et al., 2010; deWalick et al., 2012). Moreover, several studies have discovered that schistosomes showed retarded growth, development, and reproduction in immunodeficient mammalian hosts, leading to attenuated pathogenesis to hosts with decreased ovulation, such as attenuated hepatic granulomas and fibrosis formation (Amiri et al., 1992; Davies et al., 2004; Cheng et al., 2008; Tang et al., 2013). We then ask how the immunodeficiency of SCID mice could lead to the retarded growth and development of the schistosomes residing in them and what the significant changes in the gene expression of worms under their aberrant morphological changes are. However, few studies are available investigating the molecular regulation of schistosomes that is involved in their abnormal growth and development in immunodeficient hosts, although some studies focusing on the host’s factors are available (Davies et al., 2001; Blank et al., 2006; Lamb et al., 2010).

Numerous researchers had endeavored to explore the genes participating or involved in the regulation of the development and reproductive maturation of schistosomes by comparative transcriptome, proteome, and metabolome research and gained great discoveries in the growth and reproductive biology of schistosomes (Gupta and Basch, 1987; Haseeb et al., 1989; Siegel and Tracy, 1989; Haseeb and Eveland, 1991; Grevelding et al., 1997; Haseeb, 1998; Liu et al., 2006; Cogswell et al., 2012; Leutner et al., 2013; Protasio et al., 2013; Sun et al., 2014; Han et al., 2015a, b; Cao et al., 2016; Picard et al., 2016; Zhai et al., 2018; Li et al., 2019; Mao et al., 2019). Recently, we have also investigated and reported the differential metabolomic profiles of *S. japonicum* parasites from SCID and BALB/c mice in order to explore the molecular events involved in the aberrant morphologies in the growth and reproduction of schistosomes from a metabolomic level. Some important metabolic clues to what is involved in the morphological abnormalities in the growth and reproductive development of schistosomes from SCID mice have been discovered, such as the potential influence of the meiotic process due to a disturbed retinol metabolism, which is involved in meiosis initiation (Liu et al., 2019). A comprehensive understanding of the gene expression alterations would also help us find more clues about the molecular basis associated with the morphological alterations of *S. japonicum* parasites from SCID mice. Synchronously, here, we further explored and report the differentially expressed transcriptomes of *S. japonicum* parasites from SCID and BALB/c mice by RNA sequencing (RNA-seq) (Wang et al., 2009). We have identified some differentially expressed genes with higher expression levels in the gonads of the worms, i.e., ovary and vitellaria of female worms, and testes of male worms, suggesting their potentially great biological significance in the development and reproduction of schistosomes. Moreover, we also report the double-stranded RNA (dsRNA)-mediated RNA interference (RNAi) *in vivo* of the mitochondrial prohibitin complex protein 1 (*Phb1*) of the worms. *S. japonicum Phb1* was found highly expressed in the vitellaria of female worms and was found essential for the growth and reproductive development of both male and female *S. japonicum* worms.

These results, to our knowledge, provide a wealth of information on the differential gene expression profiles of schistosomes from SCID mice when compared with those from BALB/c mice. These findings also provide a rich and valuable resource for schistosomes and schistosomiasis research, such as exploring potential anti-schistosome drug and vaccine targets.

## Materials and Methods

### Ethics Statement

All experiments using *S. japonicum* parasites, *Oncomelania hupensis* (*O. hupensis*) snails, and mice were performed under protocols approved by the Institutional Animal Care and Use Committee (IACUC) at Wuhan University Center for Animal Experiments (WUCAE) according to the Regulations for the Administration of Affairs Concerning Experimental Animals of China, with ethical approval numbers 2016025, 2019098, and 2019099.

### Parasite Collection for RNA-seq

*Schistosoma japonicum*-infected *O. hupensis* were purchased from the Institute of Parasitic Disease Control and Prevention, Hunan Province, China. Six- to 7-week-old female BALB/c and SCID mice in SPF grade were purchased from Beijing Hua Fu Kang BioScience Co., Ltd. through WUCAE. The mice were maintained by WUCAE and were allowed to stay at a room temperature of 25°C with free access to food and water for 12 days in order to adapt to the new environment. The release of *S. japonicum* cercariae, artificially infecting mice, and the collection of worms on the 35th day post-infection were performed according to the methods described in our previously published article (Liu et al., 2019). After being washed with phosphate-buffered saline (PBS), the female and male worms were collected separately and equal amounts of parasites (with 20 worms in each sample) were divided into several sterile cryopreservation tubes and stored in TRIzol reagent (Invitrogen, United States). The worm samples were labeled as “S_F” for the female worms from SCID mice, “S_M” for the male worms from SCID mice, “B_F” for the female worms from BALB/c mice, and “B_M” for the male worms from BALB/c mice. The worms were then stored provisionally at −80°C until further use.

### Total RNA Extraction, Library Construction, and Sequencing

Four schistosome samples (labeled as S_F, S_M, B_F, and B_M and containing 20 worms in each sample) were separately homogenized in glass homogenizers, and their total RNAs were isolated using TRIzol reagent according to the manufacturer’s instructions (Invitrogen, United States). The potential contaminating genomic DNA was removed from the RNA samples using recombinant DNase I (RNase-free) according to the manufacturer’s protocol (Takara, China). The prepared RNA samples were packed in dry ice and delivered to the Beijing Compass Biotechnology Co., Ltd. (Beijing, China) for commercial RNA-seq analysis. Before library construction, the integrity of the RNA samples was assessed qualitatively by denatured agarose gel electrophoresis and quantitatively by an Agilent 2100 Bioanalyzer, which reported no significant degradation of the total RNA samples. The purity and the quantity of the total RNA were measured by using a Nanodrop 2000 spectrophotometer (NanoDrop Technologies, Wilmington, DE, United States). RNA quality assessment was performed by a professional technician of the Beijing Compass Company.

For transcriptome library construction, 3 μg of the total RNA from each sample was used as the input material for the subsequent RNA sample preparations. Ribosomal RNA (rRNA) was depleted using the Epicentre^®^ Ribo-zero rRNA Removal Kit. Sequencing libraries were generated and RNA sequencing was performed according to the manufacturer’s protocol using the NEBNext^®^ Ultra Directional RNA Library Prep Kit for Illumina (Illumina^®^, NEB, United States). Briefly, first-strand complementary DNA (cDNA) was synthesized using a random hexamer primer and M-MuLV Reverse Transcriptase. Second-strand cDNA synthesis was subsequently performed using DNA polymerase I and RNase H. In the reaction buffer, dNTPs with dTTP were replaced by dUTP. After end repair, adenylation of the 3′ end of the DNA fragments, and adapter ligation using the NEBNext Adaptor with a hairpin-loop structure, each library was enriched by 15 cycles of PCR, after which the library fragments were purified with the AMPure XP system (Beckman Coulter, Beverly, MA, United States) in order to obtain the stranded-specific library with select cDNA fragments of the preferred 150- to 200-bp length. The quality of the libraries was assessed on an Agilent Bioanalyzer 2100 system. The libraries were quantified with a Qubit 2.0 Fluorometer (Life Technologies, Grand Island, NY, United States) and sequencing was performed on an Illumina Hiseq^TM^ 2500 platform (Illumina).

Library construction and sequencing was performed by professional technicians of the Beijing Compass Biotechnology Co., Ltd^1^.

### Data Processing

Firstly, the raw reads of the transcriptomic sequencing were separated based on the barcodes and processed to remove adapters, poly-N-containing reads (N% > 10%), and low-quality reads (quality score ≤ 20) using custom perl scripts. Because the schistosomes were obtained from experimentally infected mice, the reads were aligned to the mouse genome using Bowtie 2 (v0.12.7) and Tophat (v2.0.0) (Trapnell et al., 2009; Langmead and Salzberg, 2012; Kim et al., 2013). Non-aligned reads were considered to be *S. japonicum*-specific. Clean reads were matched to the transcriptomes of *S. japonicum* (PRJEA34885) in *WormBase ParaSite*^2^ and assembled using Trinity, with default parameters (Liu et al., 2015). Transcripts ≤ 150 bp in length were discarded. Finally, the reconstructed unigenes were annotated by blastx to public databases [the Swiss-Prot protein database, *Caenorhabditis elegans* transcriptome, *Clonorchis sinensis* transcriptome, the UniProt protein database, *S. mansoni* transcriptome, *Schistosoma haematobium* transcriptome, *S. japonicum* V4.0, and the NCBI non-redundant (nr) database] with a threshold of 10^–10^. The number of clean reads for each transcript was calculated and then normalized to fragments per kilobase of exon model per million reads (FPKM), which associates the read numbers with the gene expression levels. Corset was applied to hierarchically cluster short transcripts into long genes for the downstream analysis (Davidson and Oshlack, 2014). The assembled transcriptome was annotated by seven public databases, including Nr, Nt, Pfam^3^ (Bateman et al., 2002), KOG/COG^4^, Swiss-Prot^5^, Kyoto Encyclopedia of Genes and Genomes (KEGG^6^) (Kanehisa et al., 2008), and Gene ontology (GO^7^). The Coding–Non-coding Index (CNCI), Coding Potential Calculator (CPC), and Pfam were used to distinguish mRNAs from long non-coding RNAs (lncRNAs) (Bateman et al., 2002; Kong et al., 2007; Sun et al., 2013). CNCI profiles can effectively distinguish protein-coding and non-coding sequences independent of the known annotations by adjoining nucleotide triplets (Sun et al., 2013). CPC searches the sequences with the known protein sequence database to clarify the coding and non-coding transcripts mainly through assessing the extent and quality of the open reading frame (ORF) in a transcript (Kong et al., 2007). Each transcript can be translated in all three possible frames and the Pfam Scan (v1.3) used to identify the occurrence of any of the known protein family domains documented in the Pfam database (Bateman et al., 2002). Transcripts without coding potential were a candidate set of lncRNAs, which will be reported and discussed elsewhere.

### Profiling of Differentially Expressed Genes and Gene Annotation

The quantification of differentially expressed genes (DEGs) in each sample was calculated by Cuffdiff (v2.1.1) (Trapnell et al., 2012), and transcripts with a *P*-adjust < 0.05 were considered differentially expressed. The gene sequences were functionally annotated using Blast2GO at www.blast2go.org (Gotz et al., 2008; Liu et al., 2015), and the output provided combined graphics for the three categories of gene ontology (GO) terms: biological processes, molecular functions, and cellular components. The KEGG automated annotation server^8^ was used to assign pathway-based functional orthology to the DEGs (Moriya et al., 2007; Kanehisa et al., 2008), in which a *P-*value less than 0.05 was considered to be statistically significant. Signal peptides were predicted using the program SignalP 4.1 server^9^ employing both the neural network and hidden Markov model (Nielsen, 2017), and transmembrane helices were predicted using TMHMM 2.0^10^ (Chen et al., 2003).

### Validation of Differential Expression by qRT-PCR

A subset of the DEGs with significant biological significance from the list of differential transcriptomes was selected for further validation by quantitative reverse transcriptase PCR (qRT-PCR). The total RNA of the above four schistosome samples collected in the same experiment was isolated using TRIzol reagent (Invitrogen, United States) and treated with recombinant RNase-free DNase I (Takara, China) according to the manufacturer’s instructions. For each sample, 1 μg of the total RNA was used to synthesize the first-strand cDNA using a Reverse Transcriptase Kit (TaKaRa, Dalian, China) with oligo(dT)_18_ primers in a final volume of 20 μl, followed by dilution of the transcribed products by adding another 20 μl ddH_2_O. qRT-PCR was performed in an optical 96-well plate on *StepOne* Plus Real-Time PCR System (Applied Biosystems, Thermo Fisher Scientific, United States) using SYBR^®^ Green PCR Master Mix (TaKaRa, Dalian, China) according to the manufacturer’s instructions. Each real-time PCR reaction (in a final volume of 20 μl) contained 10 μl of 2 × SYBR^®^ Green Real-Time PCR Master Mix, 0.25 μl of each primer (10 μM, the forward and reverse primers), 1 μl of cDNA, 0.4 μl of ROX Reference dye (50×), and 8.1 μl of sterile distilled water. In parallel, for each sample, 1 μl of sterile distilled water, as the blank template, was included as the negative control. The cycling conditions included an initial denaturation and activation at 95°C for 3 min, followed by 45 cycles at 95°C for 10 s and 60°C for 20 s. All amplifications were followed by a dissociation curve analysis of the amplified products by a dissociation step (95°C for 15 s, 65°C for 10 s, and 95°C for 10 s) to confirm the amplicon specificity for each gene. Specific primers of the candidate genes were designed using the NCBI/Primer-BLAST^11^, with specific parameters set as PCR amplicon length of 100–200 bp, melting temperature (*T*_m_) of approximately 60°C, and primer pair specificity checking against the Refseq mRNA (database) of *Schistosoma* (taxid:6181) (Organism), and were commercially synthesized by Sangon Biotech (Shanghai, China) Co., Ltd. The gene expression levels were normalized with the 26S proteasome non-ATPase regulatory subunit 4 (*PSMD4*) (Liu et al., 2012), which was validated as a reliable reference gene in the transcriptomic analysis of *S. japonicum* elsewhere (Liu et al., 2012). The relative expression of the candidate genes was calculated using the 2^–ΔΔCt^ method (Livak and Schmittgen, 2001). All reactions were run in duplicate. All primers used are listed in Supplementary Table S5.

Statistical significance in the gene expression comparison was analyzed using the IBM SPSS Statistics 20 software. The relative data are expressed as the mean ± SD, and differences between groups were determined for statistical significance using Student’s *t*-test. A *P*-value of ≤0.05 was considered to be of statistical significance.

### Relative Expression of Some Differentially Expressed Genes of Important Biological Significance in Life Cycle Stages and Gonads

Another batch of *S. japonicum*-infected *O. hupensis*, also purchased from the Institute of Parasitic Disease Control and Prevention of Hunan Province, was used to release cercariae in order to infect twenty 6- to 7-week-old SPF-grade female BALB/c mice, which were purchased from Beijing Hua Fu Kang BioScience Co., Ltd. through WUCAE. The cercariae were collected to infect BALB/c mice, with 50 ± 2 cercariae per mouse, *via* percutaneous exposure according to the methods described above. Schistosome worms at 21, 28, 35, and 49 days post-infection were collected by hepato-portal perfusion of five mice each time, and the eggs at 35 and 49 days post-infection were harvested from the livers of mice by comminution and layered filtration with a nylon mesh. The worm gonads, the testes of male worms, and the ovaries and vitellaria of female worms were separated by cutting under a dissecting microscope (Supplementary Figure S5).

For total RNA isolation and examination of the expression of some DEGs of important biological significance through life cycle stages in definitive hosts and the gonads of worms, real-time PCR was performed according to the method described above.

### dsRNA-Mediated Knockdown of *SjPhb1 in vivo*

In accordance with the standard protocols for *in vivo* RNAi in adult schistosomes (Li et al., 2018), specific *SjPhb1* dsRNA of approximately 580 bp, which was designed with the online RNAi Design Tool^12^, was generated with DNA templates (Sjp_0046680) produced by PCR with specific primers that contained a T7 RNA polymerase promoter sequence at the 5′ end (*SjPhb1*-RNAiU: 5′-GCGGATCCTAATACGACTCACTATAGGGACCTACAAACTGTGAATATAACG-3′; *SjPhb1*-RNAiL: 5′-CCGCTCGAGTAATACGACTCACTATAGGGGCAGGAAGGTTAAGAAGTG-3′). This was done by *in vitro* transcription using the T7 RiboMAX Express RNAi system (Promega, United States) according to the manufacturer’s instructions. The irrelevant enhanced green fluorescent protein (EGFP) dsRNA of approximately 640 bp was also generated using pEGFP-N1 plasmids as DNA template amplification and used as the negative control (Liu et al., 2013). The concentration of dsRNA was measured in (Biofuture, United Kingdom). The size and integrity of the resulting dsRNA was confirmed by gel electrophoresis in 1.2% agarose MOPS, which was conducted to confirm that single RNA bands were of the correct size.

Fifteen infected BALB/c mice (50 ± 2 cercariae per mouse) were designed to be randomly allocated into three groups, with five mice in each group, as follows: *SjPhb1* dsRNA injection group; EGFP dsRNA injection group set as the negative group; and 0.7% NaCl injection as the blank control group. *SjPhb1* dsRNA or EGFP dsRNA (10 μg) dissolved in 300 μl of sterilized 0.7% NaCl solution was injected into the corresponding groups of infected mice immediately after infection (day 1), which were then given additional injections every 5 days until day 26 (days 6, 11, 16, 21, and 26) (Supplementary Figure S6). The blank control group of infected mice received injection with 300 μl of 0.7% NaCl at each time point mentioned above. But during the experiment process, one infected mouse assigned to the EGFP dsRNA injection group was injected with *SjPhb1* dsRNA at the first dose by mistake, so that mouse was later assigned to the *SjPhb1* dsRNA injection group and received *SjPhb1* dsRNA injection; thus, the sample size is six mice for the *SjPhb1* dsRNA injection group and four mice for the EGFP dsRNA injection group. In addition, one mouse in the 0.7% NaCl injection group suffered accidental death when immobilized for injection, so the sample size for this group is four mice.

On the 42nd day post-infection, the three groups of mice were sacrificed and worm samples were collected for recording worm count, percent of paired worms, and size measurements (worm length), and the efficiency of *SjPhb1* dsRNA-mediated specific silencing of *SjPhb1* was confirmed by qRT-PCR. The livers of mice were also collected for pathological evaluation of egg count estimation, granuloma, and fibrosis. Specifically, to examine whether *SjPhb1* (RNAi) affected the overall growth of the worms, the parasites were immobilized on ice and imaged on a LY-WN-HPCCD, 5M Pixels High-Speed HPCCD-5 Color Microscope Camera connected to a VistaVision trinocular dissecting microscope at × 10 magnification. Parasite length was measured in digital images using the Image-Pro Plus 5.0 software (Media Cybernetics, United States). Quantitative analysis of parasite length was conducted using GraphPad Prism 6.0 software. For egg counting, 0.5 g of the left lateral lobe of the liver of each mouse was pulverized and digested in 10 ml of 5% KOH solution overnight at 37°C. Eggs in 10 μl of digest in triplicate for every sample were counted under the microscope, and the number of eggs deposited in every gram of liver per couple of worms was finally calculated (Tang et al., 2013). To examine whether *SjPhb1* knockdown in worms affected the pathogenicity of the worms, hematoxylin and eosin (H&E) and Masson staining were performed on the paraffin sections of mouse liver to evaluate the egg granuloma formation and fibrosis (Tao et al., 2018; Zhu et al., 2018).

The statistical significance in the RNAi effects comparison was analyzed using the IBM SPSS Statistics 20 software. The relative data are expressed as the mean ± SD, and differences between groups were determined for statistical significance using Student’s *t*-test. A *P*-value of ≤0.05 was considered to be of statistical significance.

## Results

### Transcriptome Profiles of Schistosomes From SCID and BALB/c Mice

**FIGURE 1:**
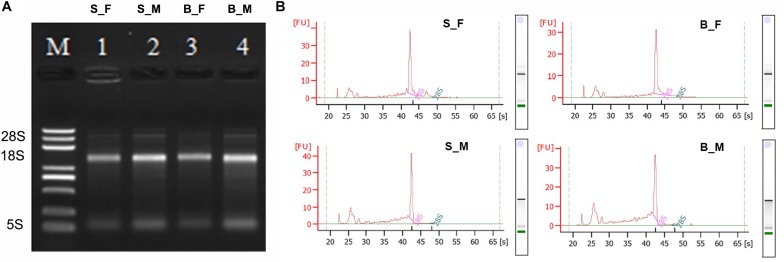
Qualitative and quantitative integrity identification of the total RNA of the *Schistosoma japonicum* worm samples. **(A)** Qualitative integrity identification of the total RNA of the *S. japonicum* worm samples by denatured agarose gel electrophoresis. Of the total RNA from each sample, 3 μl was loaded on 1.2% denaturing agarose gel with ethidium bromide (EB). The voltage of the electrophoresis was 65 V. The gel was observed and photographed with an ultraviolet transmission analyzer after the electrophoresis was finished. The 28S rRNA bands are absent, the 18S rRNA bands are present as the main bands with appropriate peaks, and the second bands are the weaker 5S bands. Therefore, the *in vivo* nick of the L-rRNA of *S. japonicum* worms total RNA was observed and confirmed here, which indicated good integrity of the total RNA of the *S. japonicum* worm samples in this study. *M*: RNA ladder. *1*: S_F; *2*: S_M; *3*: B_F; *4*: B_M. **(B)** Quantitative integrity identification of the total RNA of the *S. japonicum* worm samples by an Agilent 2100 Bioanalyzer. The peaks of 28S rRNA are also absent and the peaks of 18S rRNA are present as the main peaks for all four samples. Therefore, the *in vivo* nick of the L-rRNA in *S. japonicum* worms total RNA was also observed and confirmed here, which further indicated the good integrity and purity of the total RNA of the *S. japonicum* worm samples used here. *B_F* female worms from BALB/c mice, *B_M* male worms from BALB/c mice, *S_F* female worms from SCID mice, *S_M* male worms from SCID mice.

The four total RNA samples, including the female worms recovered from BALB/c mice (B_F), male worms recovered from BALB/c mice (B_M), female worms recovered from SCID mice (S_F), and male worms recovered from SCID mice (S_M), have prominent 18S and 5S ribosomal bands on agarose gels (Figure 1A), while the 28S rRNA band is not present due to a known gap region within the molecule (*in vivo* nick of the L-rRNA of *S. japonicum* worms total RNA) (van Keulen et al., 1991; Chen and Qiu, 1993; Li and Shi, 1997; Lu et al., 2017). According to the Bioanalyzer 2100 analysis, the 28S rRNA peak is also absent in the samples, the 18S rRNA peak is present as the main peak, and the second peak is the weaker 5S peak (Figure 1B). These indicated that all RNA samples possessed high integrity and purity and could be used for further experiments (Figure 1 and Supplementary Table S1).

A separate sequencing was performed on the double-stranded cDNA (dscDNA) libraries generated using the RNA transcripts of the 5-week-old male and female *S. japonicum* worms recovered from SCID and BALB/c mice. The Q30 was >90% for each sample (Table 1). The RNA-seq data of the transcriptomes obtained based on the total RNA of the schistosomes with high quality revealed that a total of 103.6, 142.6, 90.3, and 132.3 million raw reads were obtained in B_F, B_M, S_F, and S_M samples, respectively. And 97.6, 134.9, 84.5, and 124.6 million clear reads were obtained after quality filtration using TopHat2 (Kim et al., 2013) in B_F, B_M, S_F, and S_M samples, respectively (Table 1), in which reads matching transposon, mitochondrial, and ribosomal genes were filtered out. The mapping rates of clean reads to the reference *S. japonicum* genomic data (PRJEA34885) available at the WormBase ParaSite database^13^ (Howe et al., 2016; Lee et al., 2018) were 65.69, 61.17, 59.92, and 56.25% in B_F, B_M, S_F, and S_M samples, respectively, about 50.76, 50.67, 46.31, and 46.77% of which were uniquely mapped to the reference genome (Table 1). Of the above total, 3.51, 5.52, 3.79, and 4.54% of clean reads belonged to putative alternatively spliced fragments in B_F, B_M, S_F, and S_M samples, respectively, and the remaining clean reads corresponded to unique transcript fragments with no evidence of alternative splicing (Table 1). Approximately 20.36, 29.65, 24.13, and 25.66% of the mapped clean reads in the above four samples were mapped to the annotated genes (protein-coding genes) (Table 1), respectively.

**TABLE 1:** Statistics of the RNA-seq for each sample.

Items	B_F	B_M	S_F	S_M
Raw reads^a^	103,635,730	142,609,430	90,297,488	132,314,388
Clean reads^b^	97,638,506	134,918,766	84,544,340	124,553,760
Clean bases	14.65G	20.24G	12.68G	18.68G
Error rate (%)	0.01	0.01	0.01	0.01
Q20 (%)	97.94	97.93	97.99	97.88
Q30 (%)	94.33	94.29	94.45	94.23
GC content (%)	52.46	48.59	50.9	48.17
Total mapped (clean reads) (%)^c^	64,136,122 (65.69%)	82,527,092 (61.17%)	50,659,544 (59.92%)	70,064,375 (56.25%)
Multiple mapped (%)^d^	14,575,466 (14.93%)	14,168,401 (10.5%)	11,504,494 (13.61%)	11,805,279 (9.48%)
Uniquely mapped (%)^e^	49,560,656 (50.76%)	68,358,691 (50.67%)	39,155,050 (46.31%)	58,259,096 (46.77%)
Read-1 (%)	25,216,838 (25.83%)	34,719,994 (25.73%)	19,909,424 (23.55%)	29,584,275 (23.75%)
Read-2 (%)	24,343,818 (24.93%)	33,638,697 (24.93%)	19,245,626 (22.76%)	28,674,821 (23.02%)
Reads map to “+” (%)^f^	24,589,819 (25.18%)	33,977,708 (25.18%)	19,407,564 (22.96%)	28,942,322 (23.24%)
Reads map to “-” (%)^g^	24,970,837 (25.57%)	34,380,983 (25.48%)	19,747,486 (23.36%)	29,316,774 (23.54%)
Non-splice reads (%)^h^	46,129,379 (47.25%)	60,911,236 (45.15%)	35,949,496 (42.52%)	52,608,851 (42.24%)
Splice reads (%)^i^	3,431,277 (3.51%)	7,447,455 (5.52%)	3,205,554 (3.79%)	5,650,245 (4.54%)
Reads mapped in proper pairs (%)	39,716,752 (40.68%)	57,948,960 (42.95%)	31,646,998 (37.43%)	49,493,540 (39.74%)
Protein_coding (clean reads mapped to the annotated genes) (%)	5,329,141 (20.36%)	10,573,302 (29.65%)	5,033,590 (24.13%)	7,806,350 (25.66%)

**B_F* female worms from BALB/c mice, *B_M* male worms from BALB/c mice, *S_F* female worms from SCID mice, *S_M* male worms from SCID mice. ^*a*^Original sequencing data. ^*b*^Sampling sequence filtered by sequencing data (clean data). ^*c*^Statistics on the number of sequences that can be mapped to the genome and the number of sequences (total mapped reads) as a percentage of clean reads. ^*d*^Quantitative statistics of sequences with multiple alignment positions on the reference sequence. ^*e*^Quantitative statistics of sequences with unique alignment positions on the reference sequence. ^*f*^Statistics of the sequence alignment to the positive strands on the genome. ^*g*^Statistics of the sequences aligned to the negative strands on the genome. ^*h*^Quantitative statistics of sequences with the whole segment matched on one exon. ^*i*^Quantitative statistics of sequences fragmentally matched on two exons (also known as junction reads).*

### Sample Correlation Analysis

**FIGURE 2:**
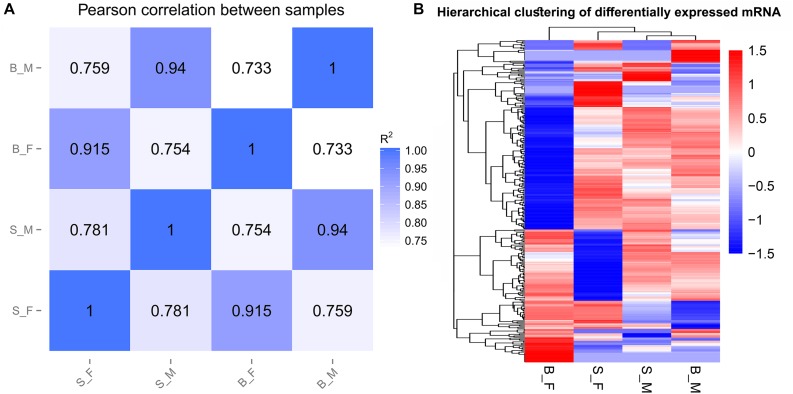
Sample relationships revealed by two different approaches. **(A)** Pearson’s correlation analysis between samples. The figures in the matrix *R*^2^ are the squares of the correlation coefficient (*r*) between two samples. **(B)** Hierarchical clustering based on the relative FPKM values [log10(FPKM + 1)] of all genes; scaling was done across all samples for each gene.

Pearson’s correlation analyses of the global expression profiles of schistosome samples revealed a stronger similarity between schistosome worms of the same sex from SCID and BALB/c mice (*R*^2^ > 0.90) than that between worms of different sexes from the same host (*R*^2^ < 0.80) (Figure 2A and Supplementary Figure S1). Similar results were obtained in the hierarchical clustering analysis of the global expression profiles of schistosome samples between the compared groups (Figure 2B). As expected, the distance of correlation between worms of the same sex from different hosts was found to be shorter than that between the worms of opposite sex from the two kinds of hosts.

### Differential Gene Expression Profiles of Schistosomes

In total, 11,183 gene transcripts were detected using FPKM > 0.01 as the threshold value by cross-referencing the *S. japonicum* gene coordinates in the WormBase ParaSite database with the coordinates of the clean reads that matched those *S. japonicum* genes (Table 2 and Supplementary Table S2). Of these, 9,993 genes were expressed in female worms from SCID mice, 10,697 were expressed in male worms from SCID mice, 9, 760 were expressed in female worms from BALB/c mice, and 10,801 genes were expressed in male worms from BALB/c mice (Table 2 and Supplementary Table S2). Moreover, 9,243 genes were commonly expressed in all four parasite forms (Table 2 and Supplementary Table S2).

**TABLE 2:** Gene expression in *Schistosoma japonicum* female and male worms recovered from SCID and BALB/c mice, respectively, as detected by RNA-seq.

Expression data summary	
Total *S. japonicum* predicted *Sjp* genes expressed	11,183
Genes expressed in S_F	9,993
Genes expressed in S_M	10,697
Genes expressed in B_F	9,760
Genes expressed in B_M	10,801
Genes commonly expressed in S_F, S_M, B_F, and B_M	9,243

**B_F* female worms from BALB/c mice, *B_M* male worms from BALB/c mice, *S_F* female worms from SCID mice, *S_M* male worms from SCID mice.*

**FIGURE 3:**
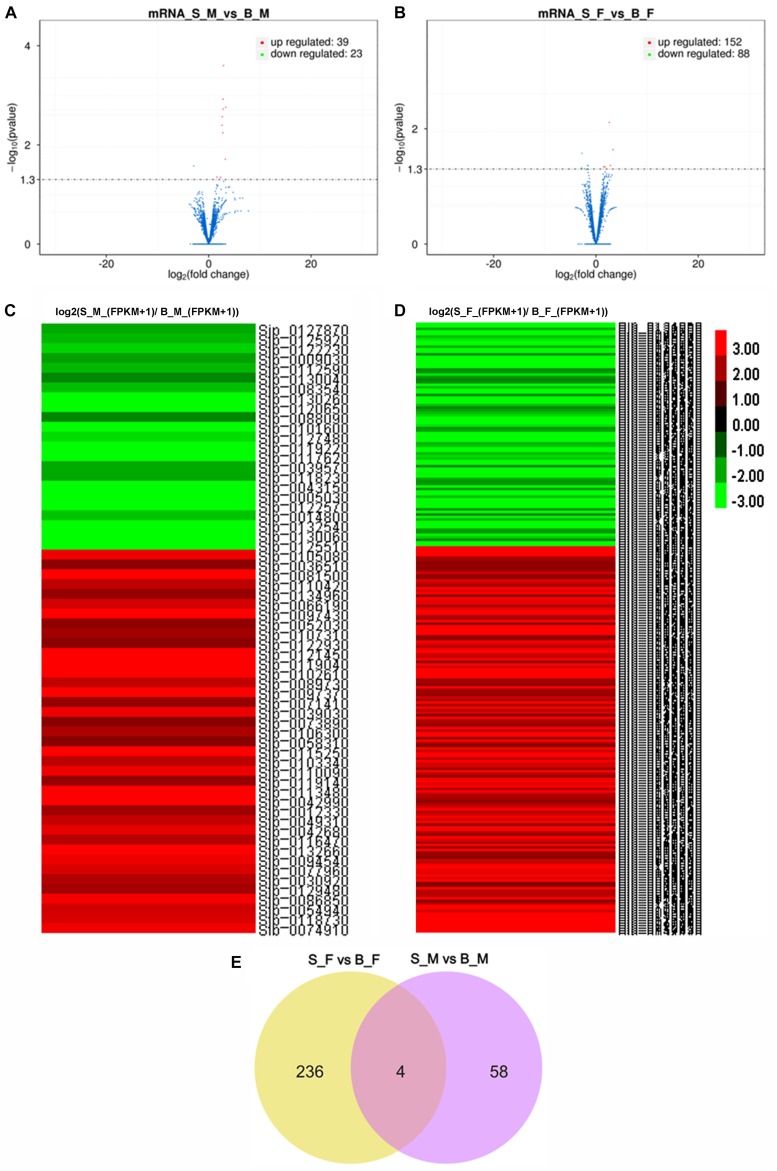
Transcription profile analysis. **(A,B)** Volcano plots of the transcriptome profiles between S_M *vs.* B_M **(A)** and between S_F *vs.* B_F **(B)**. **(C)** Heatmap of the transcriptome profiles based on the relative FPKM value ratios [log2(S_M_(FPKM + 1)/B_M_(FPKM + 1))] of all differentially expressed genes (DEGs) in S_M *vs.* B_M. **(D)** Heatmap of the transcriptome profiles based on the relative FPKM values [log2(S_F_(FPKM + 1)/B_F_(FPKM + 1))] of all DEGs in S_F *vs.* B_F.**(E)** Venn diagram of the differentially expressed genes (mRNAs) between S_F *vs.* B_F and S_M *vs.* B_M. Higher gene expression is shown in *red*, and lower gene expression is shown in *green*. *B_F* female worms from BALB/c mice, *B_M* male worms from BALB/c mice, *S_F* female worms from SCID mice, *S_M* male worms from SCID mice.

Two independent pairwise comparisons between schistosomes of the same sex recovered from SCID and BALB/c mice were performed to identify their differentially expressed genes. In detail, significant differential expression between female worms from SCID mice and female worms from BALB/c mice (S_F *vs*. B_F), and between male worms from SCID mice and male worms from BALB/c mice (S_M *vs*. B_M), was computed with a false discovery rate (FDR) of 5% and a *q* value (*P*-adjusted value) < 0.05. In brief, 298 DEGs were obtained from a pairwise comparison of the schistosome samples (i.e., S_F *vs*. B_F and S_M *vs*. B_M) between SCID mice and BALB/c mice 5 weeks post-infection. The distributions of the up- and downregulated genes between the paired comparisons are displayed as scatter plots and heatmaps (Figures 3A–D). Specifically, 62 DEGs with 39 upregulated and 23 downregulated mRNAs were found in S_M *vs*. B_M (Figure 3A and Supplementary Table S3), and 240 DEGs with 152 upregulated and 88 downregulated mRNAs were found in S_F *vs*. B_F (Figure 3B and Supplementary Table S4). It is reasonable that female worms from SCID and BALB/c mice had more differentially expressed genes than the male worms from the two hosts, which was also evidenced by the sample correlation analysis results wherein a weaker correlation was found between S_F and B_F than that between S_M and B_M (0.915 *vs*. 0.94). Of these DEGs between S_M *vs*. B_M, the top five DEGs downregulated to undetectable levels in S_M worms are Sjp_0125510 (cytochrome c oxidase subunit 2), Sjp_0130060 (cytochrome oxidase subunit I), Sjp_0117620 (26S proteasome non-ATPase regulatory subunit), Sjp_0005030 (SJCHGC08493 protein), and Sjp_0043150 (SJCHGC01077 protein); the top five DEGs upregulated in S_M worms while undetectable in B_M worms are Sjp_0121450 (histone H3.3), Sjp_0081500 (SJCHGC03331 protein), Sjp_0042990 [superoxide dismutase (Cu–Zn)], Sjp_0097430 (similar to AT-hook motif nuclear-localized protein 28-like), and Sjp_0097370 (SJCHGC01065 protein). In addition, there were 240 DEGs with 152 upregulated and 88 downregulated mRNAs for S_F *vs*. B_F (Figure 3B and Supplementary Table S4). Of these DEGs between S_F *vs*. B_F, the top five DEGs downregulated to undetectable levels in S_F worms are Sjp_0101170 (hypothetical protein), Sjp_0117410 (lactate dehydrogenase A), Sjp_0131300 ([acyl-carrier protein] *S*-malonyltransferase), Sjp_0104930 (hypothetical protein), and Sjp_0046850 (novel protein putative vertebrate RAB, member of RAS oncogene family-like 5); the top five DEGs upregulated in S_F worms while undetectable in B_F worms are Sjp_0123980 (cystinosin), Sjp_0130620 (cGMP-dependent protein kinase 1), Sjp_0134720 (putative fimbrin/plastin), Sjp_0110140 (putative pyruvate kinase), and Sjp_0077510 (putative paired box protein pax-6).

After integrating the two subsets of differentially expressed genes, moreover, a Venn diagram revealed that four DEGs are present in both comparisons (Figure 3E): they are Sjp_0117620 (26S proteasome non-ATPase regulatory subunit 10), Sjp_0127870 (mitochondrial proton/calcium exchanger protein; leucine zipper-EF-hand-containing transmembrane protein 1), Sjp_0130040 (dynein heavy chain, axonemal), and Sjp_0094540 (hypothetical protein containing proteinase inhibitor I25, cystatin domain) (Table 3). Two DEGs, Sjp_0117620 and Sjp_0094540, showed the same change direction in male and female worms from SCID mice when compared with those in worms from BALB/c mice, with the former gene downregulated and the latter gene upregulated in worms from SCID mice (Table 3). The other two DEGs, Sjp_0127870 and Sjp_0130040, showed opposite change directions in male and female worms from SCID mice, with both genes downregulated in male worms but upregulated in female worms from SCID mice (Table 3).

**TABLE 3:** Commonly present DEGs in male and female worms.

transcript_id	S_M FPKM	B_M FPKM	log2 (foldchange1)	*P* value 1	S_F FPKM	B_F FPKM	log2 (foldchange2)	*P*-value 2
Sjp_0117620	0	184.872	#NAME?	0.0009	0	212.408	#NAME?	0.0016
Sjp_0127870	0	3.051	#NAME?	0.0188	2.38326	0	inf	0.0349
Sjp_0130040	0	2.023	#NAME?	0.0188	2.34423	0	inf	0.0149
Sjp_0094540	753.542	105.928	2.8306	0.0019	263.999	43.3694	2.6058	0.0078

**B_F* female worms from BALB/c mice, *B_M* male worms from BALB/c mice, *S_F* female worms from SCID mice, *S_M* male worms from SCID mice. #NAME? was returned when S_M FPKM or S_F FPKM equals to 0 when calculating fold changes; and inf was returned when B_F FPKM or S_F FPKM equals to 0.*

### Validation of RNA-seq Data by Quantitative RT-PCR

**FIGURE 4:**
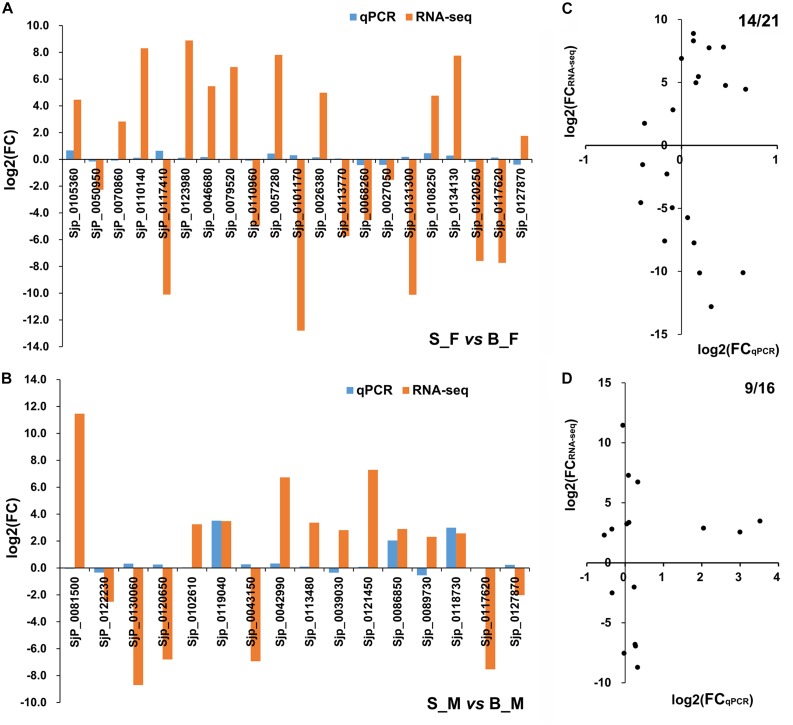
Consistency assessment of the expression patterns of 35 selected differentially expressed genes (DEGs) between RNA sequencing (RNA-seq) and quantitative reverse transcriptase PCR (qRT-PCR) data. **(A,B)** Histograms of the relative expressions of the selected 21 (19 + 2) DEGs in *Schistosoma japonicum* between S_F *vs.* B_F **(A)** and the selected 16 (14 + 2) DEGs in *S. japonicum* between S_M *vs.* B_M **(B)** using RNA-seq and qRT-PCR analysis. **(C,D)** Scatter plots for consistency assessment of the relative expressions of the 21 (19 + 2) DEGs in *S. japonicum* between S_F *vs.* B_F **(C)** and the 16 (14 + 2) DEGs in *S. japonicum* between S_M *vs.* B_M **(D)** using RNA-seq and qRT-PCR analysis. The qRT-PCR data are presented as the mean derived from duplicate experiments; their standard deviation is shown in Supplementary Figure S2. *B_F* female worms from BALB/c mice, *B_M* male worms from BALB/c mice, *S_F* female worms from SCID mice, *S_M* male worms from SCID mice.

Using 26S proteasome non-ATPase regulatory subunit 4 (*PSMD4*) as a housekeeping gene (Liu et al., 2012), a subset of 35 DEGs from both S_F *vs.* B_F and S_M *vs.* B_M comparisons were assayed by qRT-PCR to validate the data of the RNA-seq profiles (Supplementary Table S5 and Supplementary Figure S2). These selected genes were identified as DEGs in only S_F *vs.* B_F, in only S_M *vs.* B_M, or in the overlapping of both, and all of them were randomly selected among those that were annotated to ensure their biological relevance. In detail, we selected 19 DEGs just in S_F *vs.* B_F (Supplementary Figure S2A). Another two DEGs from both S_F *vs.* B_F and S_M *vs.* B_M were also selected for consideration. The RNA-seq data for the selected DEGs correlated moderately with the qPCR data in S_F *vs.* B_F, with a concordance rate of 66.67% (Figure 4C). We selected 14 DEGs just in S_M *vs.* B_M (Supplementary Figure S2B). The other two DEGs from both S_F *vs.* B_F and S_M *vs.* B_M, i.e., 26S proteasome subunit P28-related (Sjp_0117620) and leucine zipper-EF-hand-containing transmembrane protein 1, mitochondrial (Sjp_0127870), were also selected for consideration. The RNA-seq data for the selected DEGs correlated moderately with the qPCR data in S_M *vs.* B_M, with a concordance rate of 56.25% (Figure 4D).

### GO and KEGG Pathway Analyses of Differentially Expressed Genes

To identify significantly enriched gene categories and obtain a better understanding of the enriched functions of the differentially expressed genes, we performed GO term enrichment analyses (Ashburner et al., 2000). Significantly enriched GO categories (*P* < 0.05) probably identified gene groups related to parasite development and the morphological abnormalities of schistosomes from SCID mice.

**FIGURE 5:**
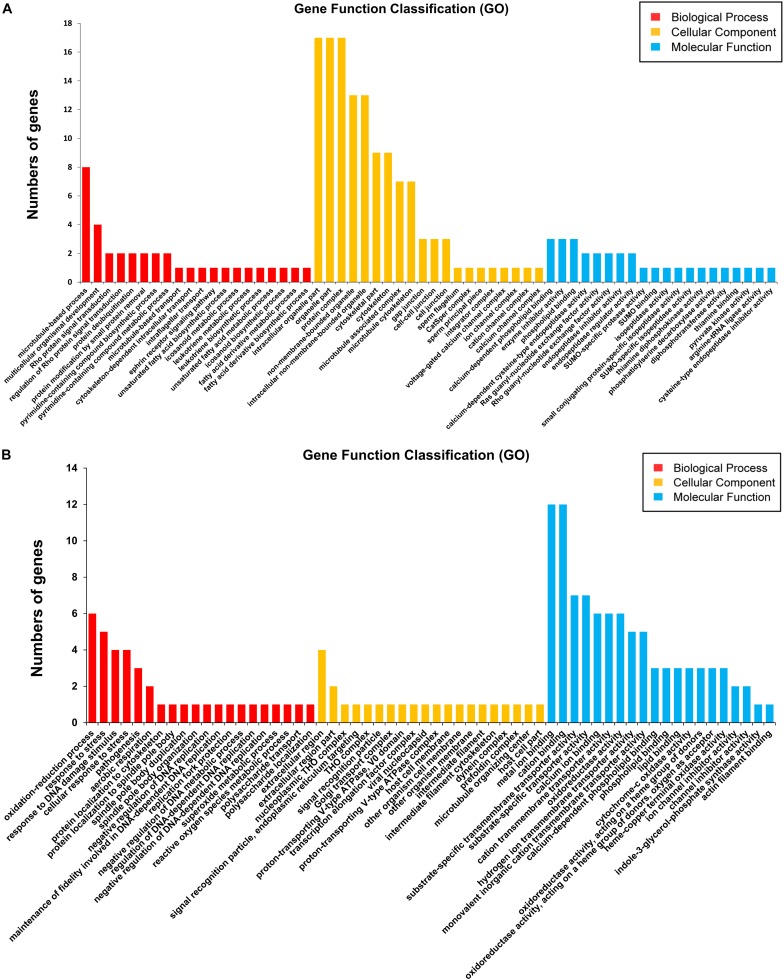
Gene ontology (GO) analysis (top 20) of the differentially expressed mRNAs in *Schistosoma japonicum* from BALB/c and SCID mice. The Blast2Go program defined the GO terms into three categories: biological process (*BP*), cellular component (*CC*), and molecular function (*MF*). Data without assigned GO terms were excluded from the graph. **(A,B)** GO analyses (top 20) of the differentially expressed mRNAs between S_F *vs.* B_F **(A)** and between S_M *vs.* B_M **(B)**. The *Y*-axis ‘*number of genes’* indicates the number of differentially expressed mRNAs between S_F *vs.* B_F or S_M *vs.* B_M, which belong to one of the indicated GO terms by GO analysis.

Firstly, 113 differentially expressed genes between S_F *vs.* B_F were annotated with GO terms in three independent categories (Supplementary Tables S4, S7). Among the biological process ontology, the top three enriched GO categories were microtubule-based process (GO:0007017), multicellular organismal development (GO:0007275), and Rho protein signal transduction (GO:0007266) (Figure 5A and Supplementary Table S7). In the case of the cellular components ontology, the top three enriched GO categories were intracellular organelle part (GO:0044446), organelle part (GO:0044422), and protein complex (GO:0043234) (Figure 5A and Supplementary Table S7). On the basis of the molecular function ontology, the top three enriched GO categories were calcium-dependent phospholipid binding (GO:0005544), enzyme inhibitor activity (GO:0004857), and phospholipid binding (GO:0005543) (Figure 5A, Supplementary Figure S3, and Supplementary Table S7). GO analysis revealed an enrichment of DEGs between S_F *vs.* B_F that are mainly involved in important biological processes and cellular components, such as microtubule-based process/microtubule-associated complex/microtubule cytoskeleton, multicellular organismal development, cytoskeletal part, cytoskeleton, which are dependent on body growth and egg formation. These important DEGs from the above categories include mainly the increased dynein light chain 1 (DLC-1, Sjp_0057280), dynein light chain 2 (DLC-2, Sjp_0008620), dynein light chain 3 (DLC-3, Sjp_0108650), tegument antigen [(I(H)A), Sjp_0045210], putative tegumental protein (Sjp_0077470), wnt5 (Sjp_0003340), chromosome segregation protein SMC (Sjp_0133570), and the decreased kinesin (Sjp_0121200), radial spoke head 10 B (Sjp_0082510), tetraspanin-CD63 receptor (Sjp_0054340), E3 ubiquitin-protein ligase PDZRN3 (Sjp_0100590), vasohibin (Sjp_0043760), and spindle assembly checkpoint component MAD1 (mitotic arrest-deficient protein 1, Sjp_0094340) (Supplementary Tables S4, S7).

Secondly, 37 differentially expressed gene sequences between S_M *vs.* B_M were annotated with GO terms in three independent categories (Supplementary Tables S3, S8). Among the biological process ontology, the top three enriched GO categories were oxidation–reduction process (GO:0055114), response to stress (GO:0006950), and response to DNA damage stimulus (GO:0006974) (Figure 5B, Supplementary Figure S4, and Supplementary Table S8). In the case of the cellular components ontology, the top three enriched GO categories were extracellular region (GO:0005576), extracellular region part (GO:0044421), and nucleoplasmic THO complex (GO:0000446) (Figure 5B and Supplementary Table S8). On the basis of the molecular function ontology, the top three enriched categories were metal ion binding (GO:0046872), cation binding (GO:0043169), and substrate-specific transmembrane transporter activity (GO:0022891) (Figure 5B and Supplementary Table S8). GO analysis also revealed an enrichment of DEGs between S_M *vs.* B_M that are mainly involved in important biological processes and cellular components, such as genes enriched in oxidation–reduction process, the decreased cytochrome c oxidase subunits 1 and 2 (cox1 and cox2) (Sjp_0130260 and Sjp_0130060), increased superoxide dismutase [Cu-Zn] (Sjp_0042990), decreased ATPase subunit 6 (Sjp_0122570), and genes enriched in response to stress or DNA damage stimulus, the increased UV excision repair protein RAD23 (Sjp_0118730) and histone H3.3 (Sjp_0121450) (Supplementary Tables S3, S8).

**FIGURE 6:**
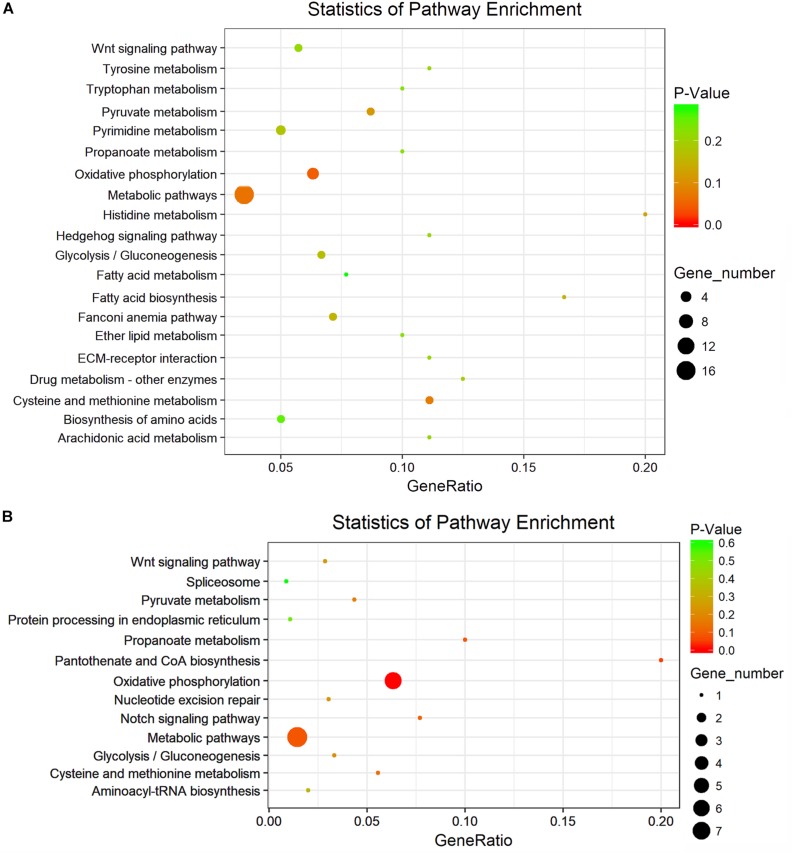
KEGG analysis with the 20 most enriched pathways. The *coloring* of the *P* value indicates the significance of the enriched factor. The *circle* indicates the target genes that are involved, and the size is proportional to the gene number. **(A,B)** KEGG analyses with the top 20 enriched pathways of the differentially expressed mRNAs in *S. japonicum* between S_F *vs.* B_F **(A)** and between S_M *vs.* B_M **(B)**.

KEGG pathway and enrichment analysis indicated that the differentially expressed genes between S_F *vs.* B_F were enriched in purine metabolism, RNA polymerase, fatty acid biosynthesis, fatty acid degradation, pyrimidine metabolism, pyruvate metabolism, fatty acid metabolism, glycolysis/gluconeogenesis, and oxidative phosphorylation (Figure 6A and Supplementary Table S9). KEGG pathway and enrichment analysis also revealed that the differentially expressed genes between S_M *vs.* B_M were enriched in oxidative phosphorylation, riboflavin metabolism, tyrosine metabolism, cysteine and methionine metabolism, propanoate metabolism, pyruvate metabolism, glycolysis/gluconeogenesis, biosynthesis of secondary metabolites, microbial metabolism in diverse environments, etc. (Figure 6B and Supplementary Table S10). Most of the enriched pathways had a *P*-value greater than 0.05 due largely to the limited number of DEG hits in the pathways.

### Expression Profiles of DEGs With Important Biological Significance in Schistosome Life Cycle Stages and Gonads

**FIGURE 7:**
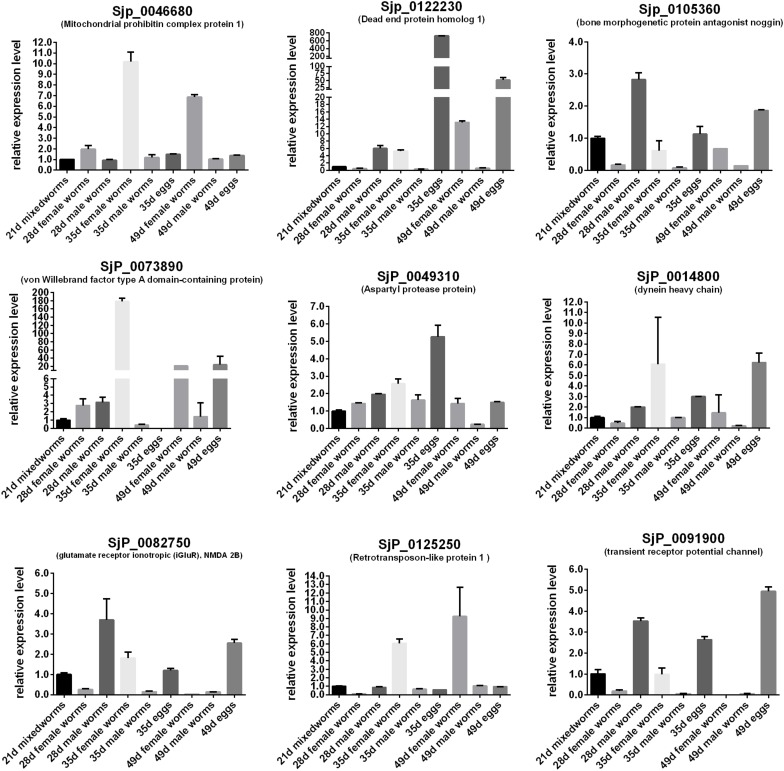
Expression profiles of some differentially expressed genes of the worms with important biological significance in reproductive and embryonic development through their life stages in BALB/c mice.

**FIGURE 8:**
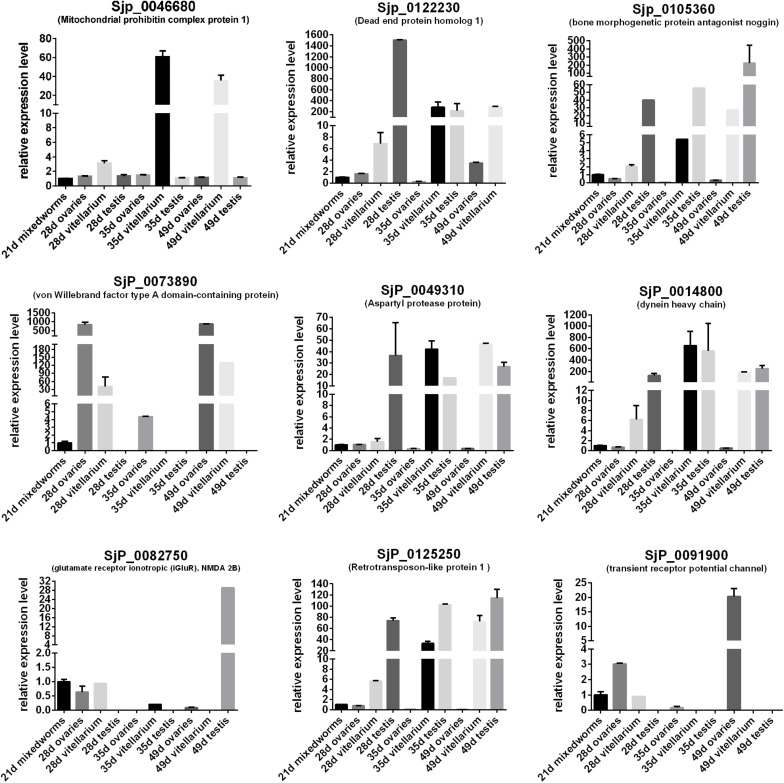
Expression profiles of some differentially expressed genes of the worms with important biological significance in the gonads (ovary, vitellaria, and testis).

In order to predict the potential important biological significance of the identified DEGs of schistosomes in their development during the life cycle stages in the definitive hosts, nine of the differentially expressed genes of important biological significance were selected for profiling their expression patterns through their life cycle in BALB/c mice (Figure 7) and in the gonads (Figure 8) by qRT-PCR. The worms, through their life cycle in mouse, were collected at 21, 28, 35, and 49 days post-infection (dpi), and the gonads (testes of the male worms and ovaries and vitelline glands of the female worms) were separated and collected from worms at 28, 35, and 49 dpi (Supplementary Figure S5). The results revealed that, firstly, mitochondrial prohibitin complex protein 1 (*Phb1*, Sjp_0046680), von Willebrand factor type A domain-containing protein (Sjp_0073890), dynein heavy chain (Sjp_0014800), and retrotransposon-like protein 1 (Sjp_0125250) were highly expressed in mature female worms at 35 and/or 49 dpi (Figure 8). Secondly, bone morphogenetic protein antagonist noggin (Sjp_0105360), glutamate receptor ionotropic (*iGluR*), NMDA 2B (Sjp_0082750), and transient receptor potential channel (Sjp_0091900) were highly expressed in male worms. Lastly, dead end protein homolog 1 (*Dnd1*, Sjp_0122230), von Willebrand factor type A domain-containing protein (Sjp_0073890), dynein heavy chain (Sjp_0014800), and transient receptor potential channel (Sjp_0091900) were highly expressed in eggs at 35 and/or 49 dpi. Additionally, *Dnd1* (Sjp_0122230), *noggin* (Sjp_0105360), aspartyl protease protein (Sjp_0049310), dynein heavy chain (Sjp_0014800), and retrotransposon-like protein 1 (Sjp_0125250) were highly expressed in the testes of male worms and the vitellaria of female worms. von Willebrand factor type A domain-containing protein (Sjp_0073890) and transient receptor potential channel (Sjp_0091900) were specifically expressed in female gonads—ovaries and/or vitellaria. Glutamate receptor ionotropic (*iGluR*), NMDA 2B (Sjp_0082750) was highly expressed in the testes of male worms.

### dsRNA-Mediated RNAi *in vivo* Revealed *SjPhb1* Is Essential to Maintain the Growth and Development of Schistosomes

In order to investigate and confirm the biological function of the identified DEGs between S_F *vs.* B_F and S_M *vs.* B_M involved in the regulation of the growth and development of schistosomes, we silenced the expression of *S. japonicum Phb1* gene (*SjPhb1*, Sjp_0046680), the function of which was predicted to be important in the development and reproduction of schistosomes, as above, using dsRNA-mediated RNAi *in vivo* through a mouse tail vein injection immediately after infection (Wang J. et al., 2017; Li et al., 2018). Prohibitin proteins are multifunctional proteins located mainly at the inner membrane of the mitochondria and expressed in universal species. Prohibitin 1 (PHB1) and prohibitin 2 (PHB2) are the two highly homologous subunits of the eukaryotic mitochondrial PHB complex. They are interdependent on the protein level, and loss of one simultaneously leads to the loss of the other. The homologous gene of prohibitin in other animals was reported to play a vital role in the mitochondria’s function, cell proteolysis, senescence, and apoptosis and is essential for embryonic viability and germline function—spermatogenesis and folliculogenesis (Artal-Sanz et al., 2003; He et al., 2008; Tyc et al., 2010; Peng et al., 2015; Chowdhury et al., 2016; Jin et al., 2016; Nguyen et al., 2016; Chai et al., 2017; Xu et al., 2017; Wang D. et al., 2017).

**FIGURE 9:**
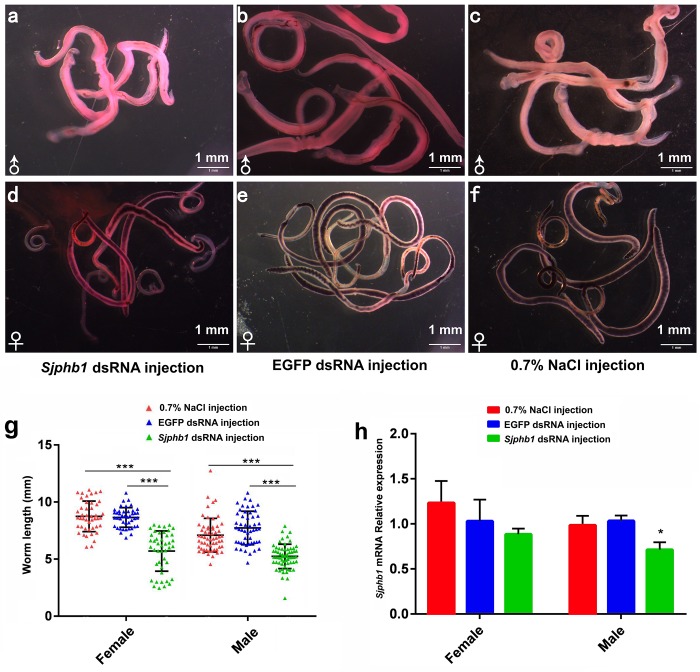
*SjPhb1* dsRNA-mediated knockdown *in vivo* negatively affected the growth of schistosome worms in their mammalian host. **(a–c)** Male worms collected at 42 days post-infection (dpi). **(d–f)** Female worms collected at 42 dpi. **(a,d)** *SjPhb1* dsRNA-treated worms. **(b,e)** EGFP dsRNA-treated worms. **(c,f)** 0.7% NaCl-treated worms. **(g)** Worm length comparison. **(h)** Efficiency assessment of the dsRNA-mediated *SjPhb1* knockdown by quantitative RT-PCR. ^∗^*P* < 0.05, ^∗∗∗^*P* < 0.001.

**FIGURE 10:**
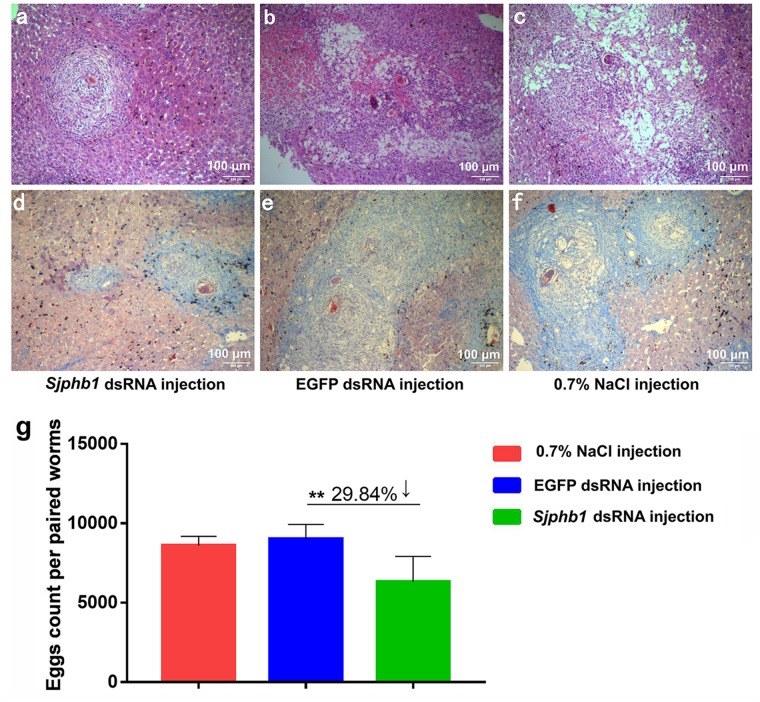
dsRNA-mediated *SjPhb1* knockdown *in vivo* resulted in a decreased egg production and attenuated granuloma formation and fibrosis in the liver of their mammalian host. **(a–c)** H&E staining of the mouse liver sections at 42 days post-infection (dpi). **(d–f)** Masson staining of the mouse liver sections at 42 dpi. **(a,d)** *SjPhb1* dsRNA injection. **(b,e)** EGFP dsRNA injection. **(c,f)** 0.7% NaCl injection. **(g)** Comparison of egg count per paired worms per gram of the liver weight of mouse. ^∗∗^*P* = 0.001.

In this experiment, BALB/c mice infected with *S. japonicum* worms were injected with specific *SjPhb1* dsRNA *via* tail vein immediately after infection, followed by an additional injection every 5 days for up to 26 dpi. The mice were finally sacrificed on day 42 after infection for effects assessment, which was in contrast to the negative (EGFP dsRNA treatment) and blank controls (0.7% NaCl treatment) (Supplementary Figure S5). No significant differences in worm burden (Supplementary Figure S7A and Supplementary Table S11) and pairing rate (Supplementary Figure S7B and Supplementary Table S11) were observed among the three groups, although a decreasing trend in worm burden could be detectable in the *SjPhb1* dsRNA treatment group (Supplementary Figure S7A and Supplementary Table S11). To our excitement, dsRNA-mediated RNAi *in vivo* led to obvious morphologic changes in worms of the specific *SjPhb1* dsRNA treatment group, wherein a significant decrease in worm length was detected in both female and male worms when compared with those of the two control groups (Figures 9a–g and Supplementary Table S11). qRT-PCR found that dsRNA treatment still led to a decrease in *SjPhb1* transcript levels by approximately 12% in female worms (*P* > 0.05) and 29% in male worms (*P* < 0.05) on the 42nd day post-infection, after a final injection on the 26th day (Figure 9h), suggesting a sustained knockdown role of *SjPhb1* dsRNA, although the dsRNA would inevitably decrease gradually since the last injection. Pathological examination of the egg granuloma and fibrosis in the livers of mice by H&E (Figures 10a–c) and Masson staining (Figures 10d–f) found a decreased size of egg granuloma and attenuated fibrosis formation in the livers of mice of the *SjPhb1* dsRNA treatment group, which could be mainly attributed to the decreased egg deposition in livers by 42.3% (Figure 10g and Supplementary Table S11).

## Discussion

In our previous publication (Tang et al., 2013), we have reported that mice lacking functional T and B cells (SCID mice) negatively influenced the growth and development of the schistosomes, which led to the downregulation of the granuloma formation in SCID mice when compared with that in BALB/c mice. Furthermore, in order to explore the molecular mechanisms involved in the regulation of growth and development of schistosomes, we also tested the metabolic profiles of the male and female *S. japonicum* worms recovered from SCID and BALB/c mice at 5 weeks post-infection using an untargeted liquid chromatography–tandem mass spectrometry (LC-MS/MS)-based high-resolution metabolomic investigation and screened out a series of perturbed metabolites and metabolic pathways potentially involved in the regulation of the growth and development of *S. japonicum* worms (Liu et al., 2019). In this study, synchronously, a comparative RNA-seq investigation was also performed to identify the distinct biosignatures in the transcriptomic profiles of male and female *S. japonicum* worms recovered from SCID and BALB/c mice, respectively.

In the results, Pearson’s correlation analyses and the hierarchical clustering analysis of the schistosome samples found a relatively weaker correlation in S_F *vs.* B_F than that in S_M *vs.* B_M. This indicates that the female schistosome worms were affected more severely than did the male worms in SCID mice when compared with those in BALB/c mice. This was also verified by the subsequent finding that more differentially expressed genes were obtained in S_F *vs*. B_F than in S_M *vs*. B_M. This could be expectable and reasonable as the growth and development of female worms were affected by male worms as well as the host’s factors, i.e., the sexual maturation of female worms depends on the pairing with male worms. A similar finding was also obtained in our previous metabolomics research on the differential metabolites in S_F *vs*. B_F than in S_M *vs*. B_M (Liu et al., 2019).

The overall results of the qRT-PCR verification of the RNA-seq data showed that more than half (23/37, 62.16%) of the tested DEGs had consistent expression patterns between the RNA-seq and qRT-PCR data (Figure 4 and Supplementary Table S6), suggesting a moderate consistency rate between the qRT-PCR and RNA-seq data. The concordance rates between the RNA-seq data and the qRT-PCR results were in the range 50–70%, which we consider to be a generally successful validation as it is already known that all transcriptome techniques, including microarray, RNA-seq, and qPCR, have their inherent pitfalls that affect accurate quantification and cannot be fully controlled (Marioni et al., 2008; Mortazavi et al., 2008; Malone and Oliver, 2011; Shi and He, 2014; Wang C. et al., 2014).

It was reported that DLC-1 of *C. elegans* and *Plasmodium falciparum* played an important role in growth and germ cell proliferation and gametogenesis *in vivo* (Daher et al., 2010; Ellenbecker et al., 2019). DLC-1 (Sjp_0057280), which is involved in microtubule-based process/microtubule-associated complex/microtubule cytoskeleton, was also identified as differentially expressed in female schistosomes from SCID mice when compared with those from BALB/c mice. This indicates a disturbed process of growth and germ cell proliferation and gametogenesis in female worms from SCID mice, which needs further research to test and confirm in the future. In addition, the enriched Rho protein signal transduction and regulation of Rho protein signal transduction (GO:0007266 and GO:0035023), with increased ephexin-1 (Sjp_0110200 and Sjp_0096350, which function as guanine nucleotide exchange factors for the important Rho-type GTPases), were also reported to be involved in a number of cell signaling pathways regulating actin cytoskeleton organization, gene transcription, cell cycle progression, and membrane trafficking (Hotchin and Hall, 1996; Tapon and Hall, 1997; Hall, 1998; Aspenstrom, 1999; Hall and Nobes, 2000; Santos et al., 2002; Vermeire et al., 2003; Quack et al., 2009; Sit and Manser, 2011; Singh et al., 2017). 5-HT affects the feeding behavior and obesity in the central nervous system. On the other hand, peripheral 5-HT may also play an important role in regulating glucose and lipid metabolism (Watanabe et al., 2014, 2016). A decrease in serotonin receptor 5HTR (Sjp_0082160) was detected in female worms from SCID mice, which suggests a weakened glucose and lipid metabolism in these worms. The protein HSP70 is an important part of the cell’s machinery for protein folding and helps protect cells from stress (Neumann et al., 1993; Maresca and Kobayashi, 1994; Jayasena et al., 1999; Brochu et al., 2004; Hatherley et al., 2014; Charnaud et al., 2017). The homolog *HSP70* (Sjp_0026380) transcript was detected as increased in female worms from SCID mice, which indicates an unfavorable environment of SCID for schistosomes. Wnt genes, encoding signaling molecules, play important roles in regulating patterning and morphogenesis during vertebrate gastrulation (Rauch et al., 1997; Kilian et al., 2003; McIntyre et al., 2013; Martyn et al., 2018). An increase of Wnt5 in female worms (Sjp_0003340) from SCID mice indicates an increased need of Wnt5 in the development of female worms from SCID mice, as SjWnt5 was predicted to play a role in the regulation of parasite muscle development and the development of the reproductive organs of both sexes based on its prominent expression in the subtegumental musculature and acetabulum musculature of schistosomula and adult worms, the testes of the male and the ovary as well as the vitellaria of the female (Ta et al., 2015). The trematode eggshell synthesis protein (Sjp_0070860) is important to the eggshell structure (Bobek et al., 1988; Ebersberger et al., 2005), the disturbance of which would result in influenced egg formation and release by female worms.

In the identified DEGs in S_M *vs.* B_M, meanwhile, cytochrome c oxidase, which is a large transformation protein complex found in the mitochondria and catalyzes the electron transport from cytochrome c to oxygen coupled to proton translocation, was reported to have important roles in energy metabolism of cell respiration and stress-induced apoptosis, and cytochrome c oxidase deficiency usually leads to muscle weakness and movement problems (Kadenbach et al., 2004; Pang et al., 2011; Douiev and Saada, 2018; Ramzan et al., 2019). Therefore, the decrease in cytochrome c oxidase in male worms from SCID mice probably results in attenuated energy metabolism and supply and increased apoptosis and finally manifests as developmental retardation or defects (Baden et al., 2007; Hatakeyama and Goto, 2017). In addition, the increase in UV excision repair protein RAD23, which is a nucleotide excision repair protein and plays an important role in nucleotide excision DNA repair through the ubiquitin-proteasome pathway when the genetic material is damaged by UV irradiation or other genotoxic stresses (Mueller and Smerdon, 1996; Schauber et al., 1998; Xie et al., 2004; Gong et al., 2006; Dantuma et al., 2009; Wade et al., 2009; Zhou et al., 2015; Lahari et al., 2017; Verma et al., 2019), also indicates potential genotoxic stresses (or unfavorable living environments) exerted by the hemato-microenvironment of SCID mice on the worms.

In summary, therefore, these differential or disturbed gene expressions associated with structural components and biological/molecular function could be inferred as the potential causes of growth and development retardation of female schistosomes from SCID mice (Anderson et al., 2015; Han et al., 2015a, b). Interestingly, furthermore, the majority of DEGs between S_F *vs.* B_F were categorized into the cellular components ontology, while most of the DEGs between S_M *vs.* B_M were categorized into the molecular function ontology (Figure 5). This phenomenon suggests that more influence or effects from the host on the female worms mainly focus on the structural composition, while more effects from the host on the male worms mainly focus on the molecular function. This could be addressed as the growth and development of the female worms being dependent on interactions with the male worms as well as their hosts (Shaw et al., 1977; Den Hollander and Erasmus, 1985; Gupta and Basch, 1987; Haseeb et al., 1989; Siegel and Tracy, 1989; Haseeb and Eveland, 1991; Kunz et al., 1995; Kunz, 2001; Hernandez et al., 2004; Han et al., 2009; deWalick et al., 2012).

In order to test and verify the biological function of the identified DEGs in the regulation of the growth and development of schistosomes, we silenced the expression of *SjPhb1* (Sjp_0046680) *in vivo* through dsRNA-mediated RNAi and confirmed that *Sjphb1* is essential in governing the parasite growth and development, although further mechanical research remains needed.

To conclude, for the first time, our current research pointed to the relevant biological processes enriched in the *S. japonicum* female and male worms from SCID mice based on their DEGs compared with those from BALB/c mice, providing a set of genes involved in the biological processes associated with the morphological abnormalities of worms in SCID mice. This research also provided a list of potential antischistosomal targets for drug or vaccine development.

## Data Availability Statement

The raw data and normalized gene-level data from the RNA-seq discussed in this article have been completely deposited in NCBI’s Gene Expression Omnibus and are accessible through GEO Series accession number GSE122317 (https://www.ncbi.nlm.nih.gov/geo/query/acc.cgi?acc=GSE122317). All relevant data are within the paper and its Supporting Information files.

## Ethics Statement

The animal study was reviewed and approved by the Institutional Animal Care and Use Committee (IACUC) at Wuhan University Center for Animal Experiments (WUCAE).

## Author Contributions

RL conceived and designed the experiments. RL, W-JC, FY, Y-DZ, Q-PZ, and H-BT performed the experiments. RL, H-FD, and HJ contributed reagents, materials, and analysis tools. RL analyzed the data and wrote the manuscript. RL and HJ critically revised the manuscript. All authors read and approved the final version of the manuscript.

## Conflict of Interest

The authors declare that the research was conducted in the absence of any commercial or financial relationships that could be construed as a potential conflict of interest.

## Footnotes

1 http://www.kangpusen.com/

2 https://parasite.wormbase.org/ftp.html

3 http://pfam.sanger.ac.uk/

4 http://www.ncbi.nlm.nih.gov/COG/

5 http://www.ebi.ac.uk/uniprot

6 http://www.genome.jp/kegg/

7 http://www.geneontology.org

8 https://www.genome.jp/kegg/

9 http://www.cbs.dtu.dk/services/SignalP/

10 http://www.cbs.dtu.dk/services/TMHMM/

11 https://www.ncbi.nlm.nih.gov/tools/primer-blast/index.cgi?LINK_LOC=BlastHome

12 https://rnaidesigner.thermofisher.com/rnaiexpress/

13 https://parasite.wormbase.org/ftp.html

## Supplementary Material

FIGURE S1 Sample relationships revealed by transcriptome profiles between worm samples. **(A–F)** Scatter plots of Pearson’s correlation analysis based on the transcriptome profiles between worm samples. The figures in the matrix R^2^ are the square of the correlation coefficient (r) between two samples.

FIGURE S2 Validation of a subset of DEGs by qRT-PCR. Thirty-five differentially expressed genes in female or male worms between SCID mice and BALB/c mice identified by RNA-Seq were selected for validation by qRT-PCR analysis. **(A)** 21 DEGs between S_F *vs.* B_F were selected for validation. **(B)** 16 DEGs between S_M *vs.* B_M were selected for validation. Bars represent standard deviation of the mean for the duplicate biological replicates. Sjp_0117620 and Sjp_0127870 were differentially expressed both S_F *vs.* B_F and S_M *vs.* B_M identified by RNA-Seq. The *PSMD4* (FN320595) was used for internal normalization. The Student’s *t*-test was used to calculate the statistical significance of the expression differences among the compared parasite groups (difference with a *P*-value < 0.05 was considered to be statistically significant).

FIGURE S3 GO analysis of the differentially expressed mRNAs between S_F vs. B_F. The Blast2Go program defined the GO terms in three categories: biological process (BP), cellular component (CC), and molecular function (MF). Data without assigned GO terms were excluded from the graph. **(A)** GO analysis of the upregulated mRNAs between S_F *vs.* B_F. **(B)** GO analysis of the downregulated mRNAs between S_F *vs.* B_F.

FIGURE S4 GO analysis of the differentially expressed mRNAs between S_M *vs.* B_M. The Blast2Go program defined the GO terms in three categories: biological process (BP), cellular component (CC), and molecular function (MF). Data without assigned GO terms were excluded from the graph. **(A)** GO analysis of the upregulated mRNAs between S_M *vs.* B_M. **(B)** GO analysis of the downregulated mRNAs between S_M *vs.* B_M.

FIGURE S5 Schematic diagram for separating testes of male worms, ovary and vitellaria of female worms by cutting under the dissecting microscope. **(1)** Anterior segment of male worm. **(2)** Testes of male worm. **(3)** Posterior segment of male worm. **(1’)** Anterior segment of female worm. **(2’)** Ovary of female worm. **(3’)** Vitellaria, posterior segment of female worm.

FIGURE S6 Schematic overview of the experimental design of RNAi *in vivo*. All biological replicates of BALB/c mice infected with *S. japonicum* cercaria (n = 5 for each group - *SjPhb1* dsRNA injection group, EGFP dsRNA injection group, and 0.7% NaCl injection group.) were maintained in the same conditions, except that they were received *SjPhb1* dsRNA injection, EGFP dsRNA injection, and 0.7% NaCl injection through tail vein immediately after infection then given an additional injection every 5 days for up to 26 days, respectively.

FIGURE S7 Worm burden and pairing rate. **(A)** Comparison of the worm burden of the three groups. **(B)** Comparison of the pairing rates of the three groups.

TABLE S1 Purity and quantity of total RNA of the *S. japonicum* worms sample.

TABLE S2 Relative expression level of detected genes among samples.

TABLE S3 Differentially expressed mRNA for S_M vs B_M and their annotation.

TABLE S4 Differentially expressed mRNA for S_F vs B_F and their annotation.

TABLE S5 Primers used in qRT-PCR for differentially expressed mRNAs validation.

TABLE S6 Data of the expression fold changes of 35 DEGs measured by RNA-seq and qRT-PCR.

TABLE S7 GO enrichment analysis for differentially expressed mRNA between S_F vs B_F_ALL. GO enrichment analysis for differentially expressed mRNA between S_F vs B_F_top20.

TABLE S8 mRNA_S_M_vs_B_M.GO_Enrichment_result_ALL. mRNA_S_M_vs_B_M.GO_Enrichment_result_TOP20.

TABLE S9 S_F_vs_B_F.DEG_KEGG_pathway_enrichment_result_ALL.

TABLE S10 S_M_vs_B_M.DEG_KEGG_pathway_enrichment_result_ALL.

TABLE S11 **(a)** dsRNA-mediated RNAi effects assessment (worm burden). **(b)** dsRNA-mediated RNAi effects assessment (pairing rate). **(c)** dsRNA-mediated RNAi effects assessment (egg count). **(d)** dsRNA-mediated RNAi effects assessment (egg count).

## References

[B1] Amiri P. Locksley R. M. Parslow T. G. Sadick M. Rector E. Ritter D. (1992). Tumour necrosis factor alpha restores granulomas and induces parasite egg-laying in schistosome-infected SCID mice. *Nature* 356 604–607. 10.1038/356604a0 1560843

[B2] Anderson L. Amaral M. S. Beckedorff F. Silva L. F. Dazzani B. Oliveira K. C. (2015). *Schistosoma mansoni* Egg, adult male and female comparative gene expression analysis and identification of novel genes by RNA-Seq. *PLoS Negl. Trop. Dis.* 9:e0004334. 10.1371/journal.pntd.0004334 26719891 PMC4699917

[B3] Artal-Sanz M. Tsang W. Y. Willems E. M. Grivell L. A. Lemire B. D. van der Spek H. (2003). The mitochondrial prohibitin complex is essential for embryonic viability and germline function in *Caenorhabditis elegans*. *J. Biol. Chem.* 278 32091–32099. 10.1074/jbc.M304877200 12794069

[B4] Ashburner M. Ball C. A. Blake J. A. Botstein D. Butler H. Cherry J. M. (2000). Gene ontology: tool for the unification of biology. *Gene Onto. Consortium. Nat. Genet.* 25 25–29. 10.1038/75556 10802651 PMC3037419

[B5] Aspenstrom P. (1999). The Rho GTPases have multiple effects on the actin cytoskeleton. *Exp. Cell Res.* 246 20–25. 10.1006/excr.1998.4300 9882511

[B6] Baden K. N. Murray J. Capaldi R. A. Guillemin K. (2007). Early developmental pathology due to cytochrome c oxidase deficiency is revealed by a new zebrafish model. *J. Biol. Chem.* 282 34839–34849. 10.1074/jbc.M703528200 17761683

[B7] Bateman A. Birney E. Cerruti L. Durbin R. Etwiller L. Eddy S. R. (2002). The Pfam protein families database. *Nucleic Acids Res.* 30 276–280. 10.1093/nar/30.1.276 11752314 PMC99071

[B8] Beckmann S. Quack T. Burmeister C. Buro C. Long T. Dissous C. (2010). *Schistosoma mansoni*: signal transduction processes during the development of the reproductive organs. *Parasitology* 137 497–520. 10.1017/S0031182010000053 20163751

[B9] Blank R. B. Lamb E. W. Tocheva A. S. Crow E. T. Lim K. C. McKerrow J. H. (2006). The common gamma chain cytokines interleukin (IL)-2 and IL-7 indirectly modulate blood fluke development via effects on CD4+ T cells. *J. Infect. Dis.* 194 1609–1616. 10.1086/508896 17083048 PMC2853799

[B10] Bobek L. A. Rekosh D. M. LoVerde P. T. (1988). Small gene family encoding an eggshell (chorion) protein of the human parasite *Schistosoma mansoni*. *Mol. Cell. Biol.* 8 3008–3016. 10.1128/mcb.8.8.3008 2850476 PMC363526

[B11] Brochu C. Haimeur A. Ouellette M. (2004). The heat shock protein HSP70 and heat shock cognate protein HSC70 contribute to antimony tolerance in the protozoan parasite leishmania. *Cell Stress Chaperones* 9 294–303. 10.1379/csc-15r1.1 15544167 PMC1065288

[B12] Cai P. Liu S. Piao X. Hou N. Gobert G. N. McManus D. P. (2016). Comprehensive transcriptome analysis of sex-biased expressed genes reveals discrete biological and physiological features of male and female *Schistosoma japonicum*. *PLoS Negl. Trop. Dis.* 10:e0004684. 10.1371/journal.pntd.0004684 27128440 PMC4851400

[B13] Cao X. Fu Z. Zhang M. Han Y. Han H. Han Q. (2016). iTRAQ-based comparative proteomic analysis of excretory-secretory proteins of schistosomula and adult worms of *Schistosoma japonicum*. *J. Proteomics* 138 30–39. 10.1016/j.jprot.2016.02.015 26915583

[B14] Chai R. R. Chen G. W. Shi H. J. O W. S. Martin-DeLeon P. A. Chen H. (2017). Prohibitin involvement in the generation of mitochondrial superoxide at complex I in human sperm. *J. Cell Mol. Med* 21 121–129. 10.1111/jcmm.12945 27558591 PMC5192824

[B15] Charnaud S. C. Dixon M. W. A. Nie C. Q. Chappell L. Sanders P. R. Nebl T. (2017). The exported chaperone Hsp70-x supports virulence functions for *Plasmodium falciparum* blood stage parasites. *PLoS One* 12:e0181656. 10.1371/journal.pone.0181656 28732045 PMC5521827

[B16] Cheever A. W. Poindexter R. W. Wynn T. A. (1999). Egg laying is delayed but worm fecundity is normal in SCID mice infected with *Schistosoma japonicum* and *S. mansoni* with or without recombinant tumor necrosis factor alpha treatment. *Infect Immun.* 67 2201–2208. 10.1128/iai.67.5.2201-2208.1999 10225875 PMC115958

[B17] Chen W. Qiu L. S. (1993). *Schistosoma japonicum* adult worm RNA isolation with Chomczynski’s method. *Zhongguo Ji Sheng Chong Xue Yu Ji Sheng Chong Bing Za Zhi* 11 135–137. 7513622

[B18] Chen Y. Yu P. Luo J. Jiang Y. (2003). Secreted protein prediction system combining CJ-SPHMM, TMHMM, and PSORT. *Mamm. Genome* 14 859–865. 10.1007/s00335-003-2296-6 14724739

[B19] Cheng Y. L. Song W. J. Liu W. Q. Lei J. H. Mo H. M. Ruppel A. (2008). The effects of T cell deficiency on the development of worms and granuloma formation in mice infected with *Schistosoma japonicum*. *Parasitol. Res.* 102 1129–1134. 10.1007/s00436-008-0880-0 18246371

[B20] Chowdhury I. Thomas K. Zeleznik A. Thompson W. E. (2016). Prohibitin regulates the FSH signaling pathway in rat granulosa cell differentiation. *J. Mol. Endocrinol.* 56 325–336. 10.1530/JME-15-0278 27044659 PMC5064770

[B21] Cogswell A. A. Kommer V. P. Williams D. L. (2012). Transcriptional analysis of a unique set of genes involved in *Schistosoma mansoni* female reproductive biology. *PLoS Negl. Trop. Dis.* 6:e1907. 10.1371/journal.pntd.0001907 23166854 PMC3499410

[B22] Daher W. Pierrot C. Kalamou H. Pinder J. C. Margos G. Dive D. (2010). *Plasmodium falciparum* dynein light chain 1 interacts with actin/myosin during blood stage development. *J. Biol. Chem.* 285 20180–20191. 10.1074/jbc.M110.102806 20421304 PMC2888431

[B23] Dantuma N. P. Heinen C. Hoogstraten D. (2009). The ubiquitin receptor Rad23: at the crossroads of nucleotide excision repair and proteasomal degradation. *DNA Repair* 8 449–460. 10.1016/j.dnarep.2009.01.005 19223247

[B24] Davidson N. M. Oshlack A. (2014). Corset: enabling differential gene expression analysis for de novo assembled transcriptomes. *Genome Biol.* 15:410. 10.1186/s13059-014-0410-6 25063469 PMC4165373

[B25] Davies S. J. Grogan J. L. Blank R. B. Lim K. C. Locksley R. M. McKerrow J. H. (2001). Modulation of blood fluke development in the liver by hepatic CD4+ lymphocytes. *Science* 294 1358–1361. 10.1126/science.1064462 11701932

[B26] Davies S. J. Lim K. C. Blank R. B. Kim J. H. Lucas K. D. Hernandez D. C. (2004). Involvement of TNF in limiting liver pathology and promoting parasite survival during schistosome infection. *Int. J. Parasitol.* 34 27–36. 10.1016/j.ijpara.2003.10.010 14711587 PMC2859728

[B27] Den Hollander J. E. Erasmus D. A. (1985). *Schistosoma mansoni*: male stimulation and DNA synthesis by the female. *Parasitology* 91(Pt 3), 449–457. 10.1017/s0031182000062697 4080418

[B28] deWalick S. Tielens A. G. van Hellemond J. J. (2012). *Schistosoma mansoni*: the egg, biosynthesis of the shell and interaction with the host. *Exp. Parasitol.* 132 7–13. 10.1016/j.exppara.2011.07.018 21840309

[B29] Douiev L. Saada A. (2018). The pathomechanism of cytochrome c oxidase deficiency includes nuclear DNA damage. *Biochim. Biophys. Acta Bioenerg.* 1859 893–900. 10.1016/j.bbabio.2018.06.004 29886046

[B30] Duan Y. Gu X. Zhu D. Sun W. Chen J. Feng J. (2014). *Schistosoma japonicum* soluble egg antigens induce apoptosis and inhibit activation of hepatic stellate cells: a possible molecular mechanism. *Int. J. Parasitol.* 44 217–224. 10.1016/j.ijpara.2013.11.003 24487000

[B31] Ebersberger I. Knobloch J. Kunz W. (2005). Cracks in the shell–zooming in on eggshell formation in the human parasite *Schistosoma mansoni*. *Dev. Genes Evol.* 215 261–267. 10.1007/s00427-005-0467-z 15747129

[B32] Ellenbecker M. Osterli E. Wang X. Day N. J. Baumgarten E. Hickey B. (2019). Dynein light chain DLC-1 facilitates the function of the germline cell fate regulator GLD-1 in *Caenorhabditis elegans*. *Genetics* 211 665–681. 10.1534/genetics.118.301617 30509955 PMC6366924

[B33] Gong F. Fahy D. Smerdon M. J. (2006). Rad4-Rad23 interaction with SWI/SNF links ATP-dependent chromatin remodeling with nucleotide excision repair. *Nat. Struct. Mol. Biol.* 13 902–907. 10.1038/nsmb1152 17013386

[B34] Gotz S. Garcia-Gomez J. M. Terol J. Williams T. D. Nagaraj S. H. Nueda M. J. (2008). High-throughput functional annotation and data mining with the Blast2GO suite. *Nucleic Acids Res.* 36 3420–3435. 10.1093/nar/gkn176 18445632 PMC2425479

[B35] Gray D. J. McManus D. P. Li Y. Williams G. M. Bergquist R. Ross A. G. (2010). Schistosomiasis elimination: lessons from the past guide the future. *Lancet Infect. Dis.* 10 733–736. 10.1016/S1473-3099(10)70099-2 20705513

[B36] Grevelding C. G. Sommer G. Kunz W. (1997). Female-specific gene expression in *Schistosoma mansoni* is regulated by pairing. *Parasitology* 115(Pt 6), 635–640. 10.1017/s0031182097001728 9488875

[B37] Gupta B. C. Basch P. F. (1987). The role of *Schistosoma mansoni* males in feeding and development of female worms. *J. Parasitol.* 73 481–486. 3298599

[B38] Hall A. (1998). Rho GTPases and the actin cytoskeleton. *Science* 279 509–514. 10.1126/science.279.5350.509 9438836

[B39] Hall A. Nobes C. D. (2000). Rho GTPases: molecular switches that control the organization and dynamics of the actin cytoskeleton. *Philos. Trans. R. Soc. Lond. B Biol. Sci.* 355 965–970. 10.1098/rstb.2000.0632 11128990 PMC1692798

[B40] Han H. Peng J. Hong Y. Fu Z. Lu K. Li H. (2015a). Comparative analysis of microRNA in schistosomula isolated from non-permissive host and susceptible host. *Mol. Biochem. Parasitol.* 204 81–88. 10.1016/j.molbiopara.2015.11.005 26844643

[B41] Han H. Peng J. Hong Y. Fu Z. Lu K. Li H. (2015b). Comparative characterization of microRNAs in *Schistosoma japonicum* schistosomula from Wistar rats and BALB/c mice. *Parasitol. Res.* 114 2639–2647. 10.1007/s00436-015-4468-1 25895062

[B42] Han Z. G. Brindley P. J. Wang S. Y. Chen Z. (2009). *Schistosoma* genomics: new perspectives on schistosome biology and host-parasite interaction. *Annu. Rev. Genomics Hum. Genet.* 10 211–240. 10.1146/annurev-genom-082908-150036 19630560

[B43] Harrison R. A. Doenhoff M. J. (1983). Retarded development of *Schistosoma mansoni* in immunosuppressed mice. *Parasitology* 86(Pt 3), 429–438. 10.1017/s0031182000050629 6877869

[B44] Haseeb M. A. (1998). *Schistosoma mansoni*: females enhance [14C]-tyrosine incorporation in males maintained in vitro. *J. Helminthol.* 72 123–126. 10.1017/s0022149x00016291 9687592

[B45] Haseeb M. A. Eveland L. K. (1991). *Schistosoma mansoni*: a chemoattractive factor released by males and its receptor in females. *Experientia* 47 970–974. 10.1007/bf01929895 1915782

[B46] Haseeb M. A. Fried B. Eveland L. K. (1989). *Schistosoma mansoni*: female-dependent lipid secretion in males and corresponding changes in lipase activity. *Int. J. Parasitol.* 19 705–709. 10.1016/0020-7519(89)90054-4 2512264

[B47] Hatakeyama H. Goto Y. I. (2017). Respiratory chain complex disorganization impairs mitochondrial and cellular integrity: phenotypic variation in cytochrome c oxidase deficiency. *Am. J. Pathol.* 187 110–121. 10.1016/j.ajpath.2016.09.003 27855277

[B48] Hatherley R. Blatch G. L. Bishop O. T. (2014). *Plasmodium falciparum* Hsp70-x: a heat shock protein at the host-parasite interface. *J. Biomol. Struct. Dyn.* 32 1766–1779. 10.1080/07391102.2013.834849 24028577

[B49] He B. Feng Q. Mukherjee A. Lonard D. M. DeMayo F. J. Katzenellenbogen B. S. (2008). A repressive role for prohibitin in estrogen signaling. *Mol. Endocrinol.* 22 344–360. 10.1210/me.2007-0400 17932104 PMC2234581

[B50] Hernandez D. C. Lim K. C. McKerrow J. H. Davies S. J. (2004). *Schistosoma mansoni*: sex-specific modulation of parasite growth by host immune signals. *Exp. Parasitol.* 106 59–61. 10.1016/j.exppara.2004.01.003 15013791 PMC2891232

[B51] Hotchin N. A. Hall A. (1996). Regulation of the actin cytoskeleton, integrins and cell growth by the Rho family of small GTPases. *Cancer Surv.* 27 311–322. 8909807

[B52] Howe K. L. Bolt B. J. Cain S. Chan J. Chen W. J. Davis P. (2016). WormBase 2016: expanding to enable helminth genomic research. *Nucleic Acids Res.* 44 D774–D780. 10.1093/nar/gkv1217 26578572 PMC4702863

[B53] Hu W. Brindley P. J. McManus D. P. Feng Z. Han Z. G. (2004). Schistosome transcriptomes: new insights into the parasite and schistosomiasis. *Trends Mol. Med.* 10 217–225. 10.1016/j.molmed.2004.03.002 15121048

[B54] Jayasena S. M. Chandrasekharan N. V. Karunanayake E. H. (1999). Molecular characterisation of a hsp70 gene from the filarial parasite *Setaria digitata*. *Int. J. Parasitol.* 29 581–591. 10.1016/s0020-7519(99)00002-8 10428634

[B55] Jin J. M. Hou C. C. Tan F. Q. Yang W. X. (2016). The potential function of prohibitin during spermatogenesis in Chinese fire-bellied newt Cynops orientalis. *Cell Tissue Res.* 363 805–822. 10.1007/s00441-015-2280-y 26384251

[B56] Kadenbach B. Arnold S. Lee I. Huttemann M. (2004). The possible role of cytochrome c oxidase in stress-induced apoptosis and degenerative diseases. *Biochim. Biophys. Acta* 1655 400–408. 10.1016/j.bbabio.2003.06.005 15100056

[B57] Kanehisa M. Araki M. Goto S. Hattori M. Hirakawa M. Itoh M. (2008). KEGG for linking genomes to life and the environment. *Nucleic Acids Res.* 36 D480–D484. 10.1093/nar/gkm882 18077471 PMC2238879

[B58] Kilian B. Mansukoski H. Barbosa F. C. Ulrich F. Tada M. Heisenberg C. P. (2003). The role of Ppt/Wnt5 in regulating cell shape and movement during zebrafish gastrulation. *Mech. Dev.* 120 467–476. 10.1016/s0925-4773(03)00004-2 12676324

[B59] Kim D. Pertea G. Trapnell C. Pimentel H. Kelley R. Salzberg S. L. (2013). TopHat2: accurate alignment of transcriptomes in the presence of insertions, deletions and gene fusions. *Genome Biol.* 14:R36. 10.1186/gb-2013-14-4-r36 23618408 PMC4053844

[B60] King C. H. Dickman K. Tisch D. J. (2005). Reassessment of the cost of chronic helmintic infection: a meta-analysis of disability-related outcomes in endemic schistosomiasis. *Lancet* 365 1561–1569. 10.1016/S0140-6736(05)66457-4 15866310

[B61] Kong L. Zhang Y. Ye Z. Q. Liu X. Q. Zhao S. Q. Wei L. (2007). CPC: assess the protein-coding potential of transcripts using sequence features and support vector machine. *Nucleic Acids Res.* 35 W345–W349. 10.1093/nar/gkm391 17631615 PMC1933232

[B62] Kunz W. (2001). Schistosome male-female interaction: induction of germ-cell differentiation. *Trends Parasitol.* 17 227–231. 10.1016/s1471-4922(01)01893-1 11323306

[B63] Kunz W. Gohr L. Grevelding C. Schussler P. Sommer G. Menrath M. (1995). *Schistosoma mansoni*: control of female fertility by the male. *Mem. Inst. Oswaldo Cruz.* 90 185–189. 10.1590/s0074-02761995000200010 8531655

[B64] Lahari T. Lazaro J. Schroeder D. F. (2017). RAD4 and RAD23/HMR Contribute to *Arabidopsis* UV Tolerance. *Genes* 9:E8. 10.3390/genes9010008 29283431 PMC5793161

[B65] Lamb E. W. Walls C. D. Pesce J. T. Riner D. K. Maynard S. K. Crow E. T. (2010). Blood fluke exploitation of non-cognate CD4+ T cell help to facilitate parasite development. *PLoS Pathog.* 6:e1000892. 10.1371/journal.ppat.1000892 20442785 PMC2861709

[B66] Langmead B. Salzberg S. L. (2012). Fast gapped-read alignment with Bowtie 2. *Nat. Methods* 9 357–359. 10.1038/nmeth.1923 22388286 PMC3322381

[B67] Lee R. Y. N. Howe K. L. Harris T. W. Arnaboldi V. Cain S. Chan J. (2018). WormBase 2017: molting into a new stage. *Nucleic Acids Res.* 46 D869–D874. 10.1093/nar/gkx998 29069413 PMC5753391

[B68] Leutner S. Oliveira K. C. Rotter B. Beckmann S. Buro C. Hahnel S. (2013). Combinatory microarray and SuperSAGE analyses identify pairing-dependently transcribed genes in *Schistosoma mansoni* males, including follistatin. *PLoS Negl. Trop. Dis.* 7:e2532. 10.1371/journal.pntd.0002532 24244773 PMC3820750

[B69] Li C. M. Shi Y. E. (1997). RNA isolation from adult *Schistosoma japonicum* by single-step method. *Shi Yong Ji Sheng Chong Bing Za Zhi* 5 165–167. 7513622

[B70] Li J. Xiang M. Zhang R. Xu B. Hu W. (2018). RNA interference in vivo in *Schistosoma japonicum*: establishing and optimization of RNAi mediated suppression of gene expression by long dsRNA in the intra-mammalian life stages of worms. *Biochem. Biophys. Res. Commun.* 503 1004–1010. 10.1016/j.bbrc.2018.06.109 29935182

[B71] Li X. Qiao H. Qin F. Cheng G. Liu J. Li H. (2019). Comparative analysis of iTRAQ-based proteome profiles of *Schistosoma japonicum* female worms coming from single-sex infections and bisexual infections. *J. Proteomics* 213:103597. 10.1016/j.jprot.2019.103597 31778827

[B72] Liu F. Lu J. Hu W. Wang S. Y. Cui S. J. Chi M. (2006). New perspectives on host-parasite interplay by comparative transcriptomic and proteomic analyses of *Schistosoma japonicum*. *PLoS Pathog.* 2:e29. 10.1371/journal.ppat.0020029 16617374 PMC1435792

[B73] Liu R. Cheng W. J. Tang H. B. Zhong Q. P. Ming Z. P. Dong H. F. (2019). Comparative metabonomic investigations of *Schistosoma japonicum* from SCID Mice and BALB/c mice: clues to developmental abnormality of schistosome in the immunodeficient host. *Front. Microbiol.* 10:440. 10.3389/fmicb.2019.00440 30915055 PMC6423161

[B74] Liu R. Zhao Q. P. Ye Q. Xiong T. Tang C. L. Dong H. F. (2013). Cloning and characterization of a bone morphogenetic protein homologue of *Schistosoma japonicum*. *Exp. Parasitol.* 135 64–71. 10.1016/j.exppara.2013.05.016 23756146

[B75] Liu S. Cai P. Hou N. Piao X. Wang H. Hung T. (2012). Genome-wide identification and characterization of a panel of house-keeping genes in *Schistosoma japonicum*. *Mol. Biochem. Parasitol.* 182 75–82. 10.1016/j.molbiopara.2011.12.007 22245333

[B76] Liu S. Zhou X. Piao X. Wu C. Hou N. Chen Q. (2015). Comparative analysis of transcriptional profiles of Adult *Schistosoma japonicum* from different laboratory animals and the natural host. Water Buffalo. *PLoS Negl. Trop. Dis.* 9:e0003993. 10.1371/journal.pntd.0003993 26285138 PMC4540470

[B77] Livak K. J. Schmittgen T. D. (2001). Analysis of relative gene expression data using real-time quantitative PCR and the 2(-Delta Delta C(T)) Method. *Methods* 25 402–408. 10.1006/meth.2001.1262 11846609

[B78] LoVerde P. T. Andrade L. F. Oliveira G. (2009). Signal transduction regulates schistosome reproductive biology. *Curr. Opin. Microbiol.* 12 422–428. 10.1016/j.mib.2009.06.005 19577949 PMC2740793

[B79] Lu Z. Sessler F. Holroyd N. Hahnel S. Quack T. Berriman M. (2016). Schistosome sex matters: a deep view into gonad-specific and pairing-dependent transcriptomes reveals a complex gender interplay. *Sci. Rep.* 6:31150. 10.1038/srep31150 27499125 PMC4976352

[B80] Lu Z. Sessler F. Holroyd N. Hahnel S. Quack T. Berriman M. (2017). A gene expression atlas of adult *Schistosoma mansoni* and their gonads. *Sci. Data* 4:170118. 10.1038/sdata.2017.118 28829433 PMC5566097

[B81] Malone J. H. Oliver B. (2011). Microarrays, deep sequencing and the true measure of the transcriptome. *BMC Biol.* 9:34. 10.1186/1741-7007-9-34 21627854 PMC3104486

[B82] Mao Y. He C. Li H. Lu K. Fu Z. Hong Y. (2019). Comparative analysis of transcriptional profiles of *Schistosoma japonicum* adult worms derived from primary-infected and re-infected water buffaloes. *Parasit Vectors* 12:340. 10.1186/s13071-019-3600-y 31296252 PMC6625002

[B83] Maresca B. Kobayashi G. S. (1994). Hsp70 in parasites: as an inducible protective protein and as an antigen. *Experientia* 50 1067–1074. 10.1007/bf01923463 7988666

[B84] Marioni J. C. Mason C. E. Mane S. M. Stephens M. Gilad Y. (2008). RNA-seq: an assessment of technical reproducibility and comparison with gene expression arrays. *Genome Res.* 18 1509–1517. 10.1101/gr.079558.108 18550803 PMC2527709

[B85] Martyn I. Kanno T. Y. Ruzo A. Siggia E. D. Brivanlou A. H. (2018). Self-organization of a human organizer by combined Wnt and Nodal signalling. *Nature* 558 132–135. 10.1038/s41586-018-0150-y 29795348 PMC6077985

[B86] McIntyre D. C. Seay N. W. Croce J. C. McClay D. R. (2013). Short-range Wnt5 signaling initiates specification of sea urchin posterior ectoderm. *Development* 140 4881–4889. 10.1242/dev.095844 24227654 PMC3848187

[B87] Morais S. B. Figueiredo B. C. Assis N. R. G. Alvarenga D. M. de Magalhaes M. T. Q. Ferreira R. S. (2018). *Schistosoma mansoni* SmKI-1 serine protease inhibitor binds to elastase and impairs neutrophil function and inflammation. *PLoS Pathog.* 14:e1006870. 10.1371/journal.ppat.1006870 29425229 PMC5823468

[B88] Moriya Y. Itoh M. Okuda S. Yoshizawa A. C. Kanehisa M. (2007). KAAS: an automatic genome annotation and pathway reconstruction server. *Nucleic Acids Res.* 35 W182–W185. 10.1093/nar/gkm321 17526522 PMC1933193

[B89] Mortazavi A. Williams B. A. McCue K. Schaeffer L. Wold B. (2008). Mapping and quantifying mammalian transcriptomes by RNA-Seq. *Nat. Methods* 5 621–628. 10.1038/nmeth.1226 18516045 PMC13303166

[B90] Mueller J. P. Smerdon M. J. (1996). Rad23 is required for transcription-coupled repair and efficient overrall repair in *Saccharomyces cerevisiae*. *Mol. Cell. Biol.* 16 2361–2368. 10.1128/mcb.16.5.2361 8628303 PMC231224

[B91] Nakazawa M. Fantappie M. R. Freeman G. L. Jr. Eloi-Santos S. Olsen N. J. Kovacs W. J. (1997). *Schistosoma mansoni*: susceptibility differences between male and female mice can be mediated by testosterone during early infection. *Exp. Parasitol.* 85 233–240. 10.1006/expr.1997.4148 9085920

[B92] Neumann S. Ziv E. Lantner F. Schechter I. (1993). Regulation of HSP70 gene expression during the life cycle of the parasitic helminth *Schistosoma mansoni*. *Eur. J. Biochem.* 212 589–596. 10.1111/j.1432-1033.1993.tb17697.x 8444195

[B93] Nguyen K. H. Ande S. R. Mishra S. (2016). Prohibitin: an unexpected role in sex dimorphic functions. *Biol. Sex. Differ.* 7:30. 10.1186/s13293-016-0083-9 27347368 PMC4921003

[B94] Nielsen H. (2017). Predicting secretory proteins with SignalP. *Methods Mol. Biol.* 1611 59–73. 10.1007/978-1-4939-7015-5_6 28451972

[B95] Pang L. Qiu T. Cao X. Wan M. (2011). Apoptotic role of TGF-beta mediated by Smad4 mitochondria translocation and cytochrome c oxidase subunit II interaction. *Exp. Cell Res.* 317 1608–1620. 10.1016/j.yexcr.2011.02.004 21324314

[B96] Peng Y. T. Chen P. Ouyang R. Y. Song L. (2015). Multifaceted role of prohibitin in cell survival and apoptosis. *Apoptosis* 20 1135–1149. 10.1007/s10495-015-1143-z 26091791 PMC4531144

[B97] Picard M. A. Boissier J. Roquis D. Grunau C. Allienne J. F. Duval D. (2016). Sex-Biased Transcriptome of *Schistosoma mansoni*: host-parasite interaction, genetic determinants and epigenetic regulators are associated with sexual differentiation. *PLoS Negl. Trop Dis.* 10:e0004930. 10.1371/journal.pntd.0004930 27677173 PMC5038963

[B98] Platt T. R. Brooks D. R. (1997). Evolution of the schistosomes (*Digenea*: *Schistosomatoidea*): the origin of dioecy and colonization of the venous system. *J. Parasitol.* 83 1035–1044. 9406775

[B99] Protasio A. V. Dunne D. W. Berriman M. (2013). Comparative study of transcriptome profiles of mechanical- and skin-transformed *Schistosoma mansoni* schistosomula. *PLoS Negl. Trop. Dis.* 7:e2091. 10.1371/journal.pntd.0002091 23516644 PMC3597483

[B100] Quack T. Beckmann S. Grevelding C. G. (2006). Schistosomiasis and the molecular biology of the male-female interaction of *S. mansoni*. *Berl. Munch. Tierarztl. Wochenschr.* 119 365–372. 17007463

[B101] Quack T. Knobloch J. Beckmann S. Vicogne J. Dissous C. Grevelding C. G. (2009). The formin-homology protein SmDia interacts with the Src kinase SmTK and the GTPase SmRho1 in the gonads of *Schistosoma mansoni*. *PLoS One* 4:e6998. 10.1371/journal.pone.0006998 19746159 PMC2734992

[B102] Ramzan R. Rhiel A. Weber P. Kadenbach B. Vogt S. (2019). Reversible dimerization of cytochrome c oxidase regulates mitochondrial respiration. *Mitochondrion* 49 149–155. 10.1016/j.mito.2019.08.002 31419492

[B103] Rauch G. J. Hammerschmidt M. Blader P. Schauerte H. E. Strahle U. Ingham P. W. (1997). Wnt5 is required for tail formation in the zebrafish embryo. *Cold. Spring Harb. Symp. Quant. Biol.* 62 227–234. 9598355

[B104] Roderfeld M. Padem S. Lichtenberger J. Quack T. Weiskirchen R. Longerich T. (2018). *Schistosoma mansoni* egg secreted antigens activate HCC-associated transcription factors c-Jun and STAT3 in hamster and human hepatocytes. *Hepatology* 27 10.1002/hep.30192 30053321 PMC7496692

[B105] Ross A. G. Bartley P. B. Sleigh A. C. Olds G. R. Li Y. Williams G. M. (2002). Schistosomiasis. *N. Engl. J. Med.* 346 1212–1220. 10.1056/NEJMra012396 11961151

[B106] Ross A. G. Olveda R. M. Acosta L. Harn D. A. Chy D. Li Y. (2013). Road to the elimination of schistosomiasis from Asia: the journey is far from over. *Microbes Infect.* 15 858–865. 10.1016/j.micinf.2013.07.010 23973709 PMC4433715

[B107] Santos T. M. Machado C. R. Franco G. R. Pena S. D. (2002). Characterization and comparative functional analysis in yeast of a *Schistosoma mansoni* Rho1 GTPase gene. *Mol. Biochem. Parasitol.* 125 103–112. 10.1016/s0166-6851(02)00218-9 12467978

[B108] Schauber C. Chen L. Tongaonkar P. Vega I. Lambertson D. Potts W. (1998). Rad23 links DNA repair to the ubiquitin/proteasome pathway. *Nature* 391 715–718. 10.1038/35661 9490418

[B109] Shaw J. R. Marshall I. Erasmus D. A. (1977). *Schistosoma mansoni*: in vitro stimulation of vitelline cell development by extracts of male worms. *Exp. Parasitol.* 42 14–20. 10.1016/0014-4894(77)90056-x 862702

[B110] Shi Y. He M. (2014). Differential gene expression identified by RNA-Seq and qPCR in two sizes of pearl oyster (*Pinctada fucata*). *Gene* 538 313–322. 10.1016/j.gene.2014.01.031 24440293

[B111] Siegel D. A. Tracy J. W. (1989). *Schistosoma mansoni*: influence of the female parasite on glutathione biosynthesis in the male. *Exp. Parasitol.* 69 116–124. 10.1016/0014-4894(89)90179-3 2753119

[B112] Singh V. Davidson A. C. Hume P. J. Humphreys D. Koronakis V. (2017). Arf GTPase interplay with Rho GTPases in regulation of the actin cytoskeleton. *Small GTPases* 10 411–418. 10.1080/21541248.2017.1329691 28524754 PMC6748364

[B113] Sit S. T. Manser E. (2011). Rho GTPases and their role in organizing the actin cytoskeleton. *J. Cell Sci.* 124(Pt 5), 679–683. 10.1242/jcs.064964 21321325

[B114] Steinmann P. Keiser J. Bos R. Tanner M. Utzinger J. (2006). Schistosomiasis and water resources development: systematic review, meta-analysis, and estimates of people at risk. *Lancet Infect. Dis.* 6 411–425. 10.1016/S1473-3099(06)70521-7 16790382

[B115] Sun J. Wang S. Li C. Ren Y. Wang J. (2014). Novel expression profiles of microRNAs suggest that specific miRNAs regulate gene expression for the sexual maturation of female *Schistosoma japonicum* after pairing. *Parasit Vectors* 7:177. 10.1186/1756-3305-7-177 24721600 PMC4021575

[B116] Sun L. Luo H. Bu D. Zhao G. Yu K. Zhang C. (2013). Utilizing sequence intrinsic composition to classify protein-coding and long non-coding transcripts. *Nucleic Acids Res.* 41:e166. 10.1093/nar/gkt646 23892401 PMC3783192

[B117] Ta N. Feng X. Deng L. Fu Z. Hong Y. Liu J. (2015). Characterization and expression analysis of Wnt5 in *Schistosoma japonicum* at different developmental stages. *Parasitol. Res.* 114 3261–3269. 10.1007/s00436-015-4545-5 26077755

[B118] Tang H. Ming Z. Liu R. Xiong T. Grevelding C. G. Dong H. (2013). Development of adult worms and granulomatous pathology are collectively regulated by T- and B-cells in mice infected with *Schistosoma japonicum*. *PLoS One* 8:e54432. 10.1371/journal.pone.0054432 23349889 PMC3551845

[B119] Tao R. Fan X. X. Yu H. J. Ai G. Zhang H. Y. Kong H. Y. (2018). MicroRNA-29b-3p prevents *Schistosoma japonicum*-induced liver fibrosis by targeting COL1A1 and COL3A1. *J. Cell. Biochem.* 119 3199–3209. 10.1002/jcb.26475 29091295

[B120] Tapon N. Hall A. (1997). Rho, Rac and Cdc42 GTPases regulate the organization of the actin cytoskeleton. *Curr. Opin. Cell Biol.* 9 86–92. 10.1016/s0955-0674(97)80156-1 9013670

[B121] The Schistosoma japonicum Genome Sequencing and Functional Analysis Consortium (2009). The *Schistosoma japonicum* genome reveals features of host-parasite interplay. *Nature* 460 345–351. 10.1038/nature08140 19606140 PMC3747554

[B122] Trapnell C. Pachter L. Salzberg S. L. (2009). TopHat: discovering splice junctions with RNA-Seq. *Bioinformatics* 25 1105–1111. 10.1093/bioinformatics/btp120 19289445 PMC2672628

[B123] Trapnell C. Roberts A. Goff L. Pertea G. Kim D. Kelley D. R. (2012). Differential gene and transcript expression analysis of RNA-seq experiments with TopHat and Cufflinks. *Nat. Protoc.* 7 562–578. 10.1038/nprot.2012.016 22383036 PMC3334321

[B124] Tyc J. Faktorova D. Kriegova E. Jirku M. Vavrova Z. Maslov D. A. (2010). Probing for primary functions of prohibitin in *Trypanosoma brucei*. *Int. J. Parasitol.* 40 73–83. 10.1016/j.ijpara.2009.07.008 19683530

[B125] van Keulen H. Mertz P. M. LoVerde P. T. Shi H. Rekosh D. M. (1991). Characterization of a 54-nucleotide gap region in the 28S rRNA gene of *Schistosoma mansoni*. *Mol. Biochem. Parasitol.* 45 205–214. 10.1016/0166-6851(91)90087-m 2038356

[B126] Verma S. Shakya V. P. S. Idnurm A. (2019). The dual function gene RAD23 contributes to *Cryptococcus neoformans* virulence independently of its role in nucleotide excision DNA repair. *Gene* 717:144043. 10.1016/j.gene.2019.144043 31400407

[B127] Vermeire J. J. Osman A. LoVerde P. T. Williams D. L. (2003). Characterisation of a Rho homologue of *Schistosoma mansoni*. *Int. J. Parasitol.* 33 721–731. 10.1016/s0020-7519(03)00046-8 12814652

[B128] Wade S. L. Poorey K. Bekiranov S. Auble D. T. (2009). The Snf1 kinase and proteasome-associated Rad23 regulate UV-responsive gene expression. *EMBO J.* 28 2919–2931. 10.1038/emboj.2009.229 19680226 PMC2760106

[B129] Wang C. Gong B. Bushel P. R. Thierry-Mieg J. Thierry-Mieg D. Xu J. (2014). The concordance between RNA-seq and microarray data depends on chemical treatment and transcript abundance. *Nat. Biotechnol.* 32 926–932. 10.1038/nbt.3001 25150839 PMC4243706

[B130] Wang J. Xu F. Zhu D. Duan Y. Chen J. Sun X. (2014). *Schistosoma japonicum* soluble egg antigens facilitate hepatic stellate cell apoptosis by downregulating Akt expression and upregulating p53 and DR5 expression. *PLoS Negl. Trop. Dis.* 8:e3106. 10.1371/journal.pntd.0003106 25144704 PMC4140669

[B131] Wang D. Zhao Y. Q. Han Y. L. Hou C. C. Zhu J. Q. (2017). Characterization of mitochondrial prohibitin from *Boleophthalmus pectinirostris* and evaluation of its possible role in spermatogenesis. *Fish. Physiol. Biochem.* 43 1299–1313. 10.1007/s10695-017-0373-0 28501977

[B132] Wang J. Yu Y. Shen H. Qing T. Zheng Y. Li Q. (2017). Dynamic transcriptomes identify biogenic amines and insect-like hormonal regulation for mediating reproduction in *Schistosoma japonicum*. *Nat. Commun.* 8:14693. 10.1038/ncomms14693 28287085 PMC5355954

[B133] Wang Z. Gerstein M. Snyder M. (2009). RNA-Seq: a revolutionary tool for transcriptomics. *Nat. Rev. Genet.* 10 57–63. 10.1038/nrg2484 19015660 PMC2949280

[B134] Watanabe H. Nakano T. Saito R. Akasaka D. Saito K. Ogasawara H. (2016). Serotonin improves high fat diet induced obesity in mice. *PLoS One* 11:e0147143. 10.1371/journal.pone.0147143 26766570 PMC4713156

[B135] Watanabe H. Saito R. Nakano T. Takahashi H. Takahashi Y. Sumiyoshi K. (2014). Effect of peripheral 5-HT on glucose and lipid metabolism in wether sheep. *PLoS One* 9:e88058. 10.1371/journal.pone.0088058 24505376 PMC3913723

[B136] WHO (2016). Schistosomiasis: number of people treated worldwide in 2014. *Wkly Epidemiol. Rec.* 91 53–60. 26852429

[B137] Xie Z. Liu S. Zhang Y. Wang Z. (2004). Roles of Rad23 protein in yeast nucleotide excision repair. *Nucleic Acids Res.* 32 5981–5990. 10.1093/nar/gkh934 15545636 PMC534619

[B138] Xu Y. R. Fan Y. S. Yang W. X. (2017). Mitochondrial prohibitin and its ubiquitination during spermatogenesis of the swimming crab *Charybdis japonica*. *Gene* 627 137–148. 10.1016/j.gene.2017.06.025 28627439

[B139] Zhai Q. Fu Z. Hong Y. Yu X. Han Q. Lu K. (2018). iTRAQ-based comparative proteomic analysis of Adult *Schistosoma japonicum* from water buffalo and yellow cattle. *Front. Microbiol.* 9:99. 10.3389/fmicb.2018.00099 29467732 PMC5808103

[B140] Zhou Z. Humphryes N. van Eijk P. Waters R. Yu S. Kraehenbuehl R. (2015). UV induced ubiquitination of the yeast Rad4-Rad23 complex promotes survival by regulating cellular dNTP pools. *Nucleic Acids Res.* 43 7360–7370. 10.1093/nar/gkv680 26150418 PMC4551923

[B141] Zhu Y. Ni Y. Liu R. Hou M. Yang B. Song J. (2018). PPAR-gamma agonist alleviates liver and spleen pathology via inducing treg cells during *Schistosoma japonicum* infection. *J. Immunol. Res.* 2018:6398078. 10.1155/2018/6398078 30116754 PMC6079474

