# Supplementary figures and images for: Comparative Transcriptome Analyses of Schistosoma japonicum Derived From SCID Mice and BALB/c Mice: Clues to the Abnormality in Parasite Growth and Development
Source: Front Microbiol. 2020 Mar 11;11:274. doi: 10.3389/fmicb.2020.00274 (PMC7078119; doi:10.3389/fmicb.2020.00274)

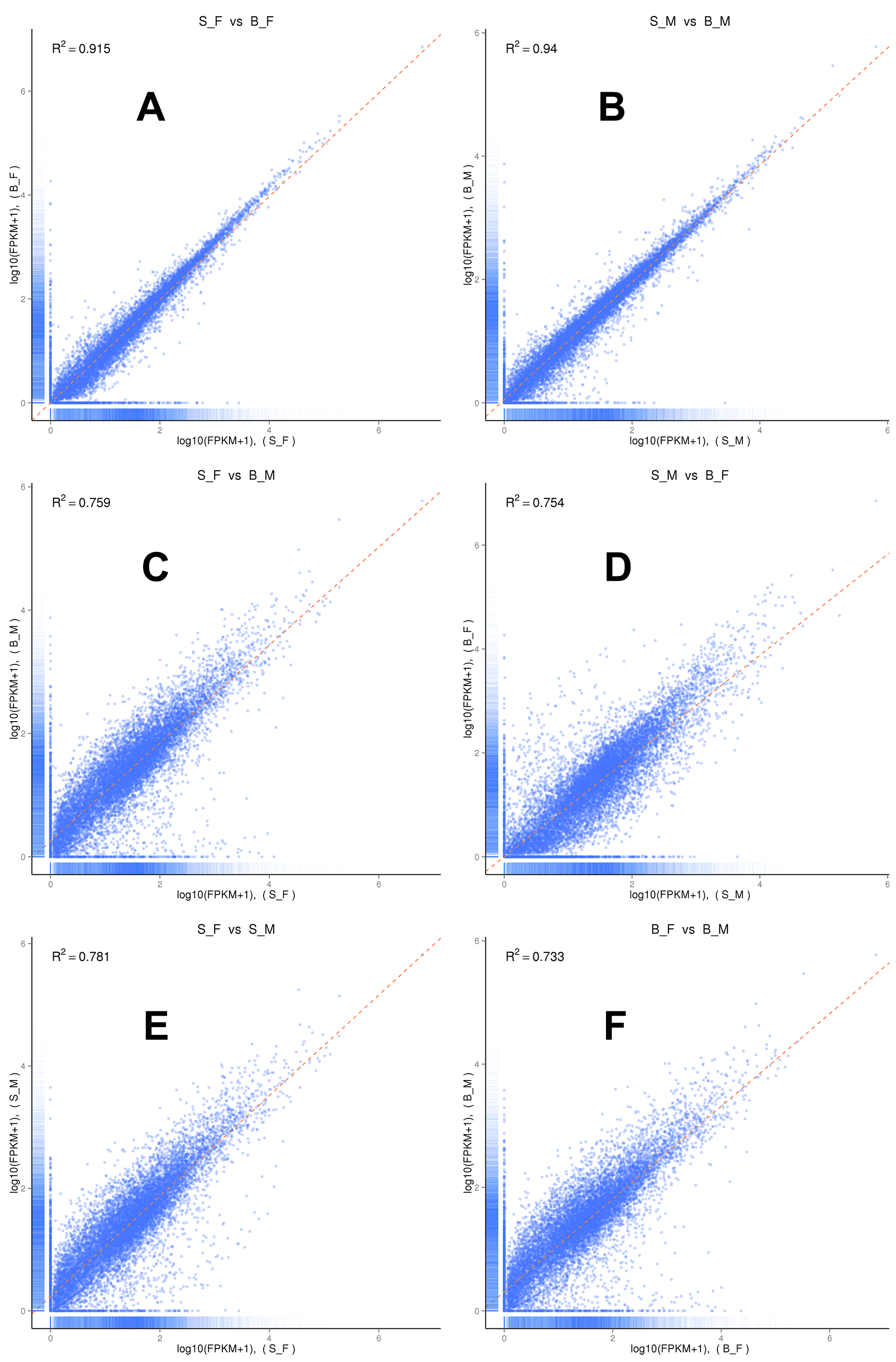

Supplement: FIGURE S1 — Sample relationships revealed by transcriptome profiles between worm samples. (A–F) Scatter plots of Pearson’s correlation analysis based on the transcriptome profiles between worm samples. The figures in the matrix R2 are the square of the correlation coefficient (r) between two samples. [file Data_Sheet_1.zip › Data Sheet 1/Figure S1.tif]

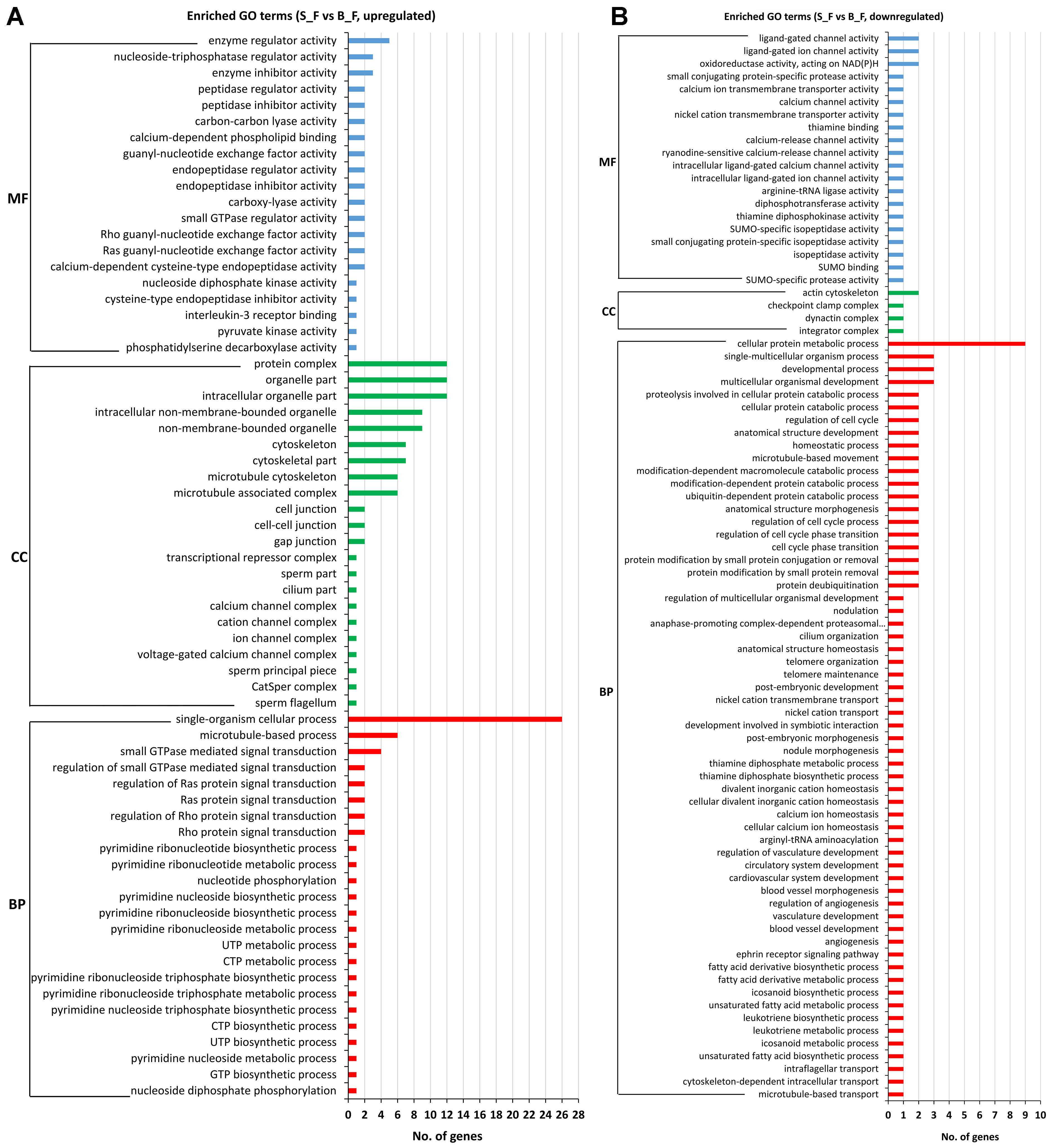

Supplement: FIGURE S1 — Sample relationships revealed by transcriptome profiles between worm samples. (A–F) Scatter plots of Pearson’s correlation analysis based on the transcriptome profiles between worm samples. The figures in the matrix R2 are the square of the correlation coefficient (r) between two samples. [file Data_Sheet_1.zip › Data Sheet 1/Figure S3.tif]

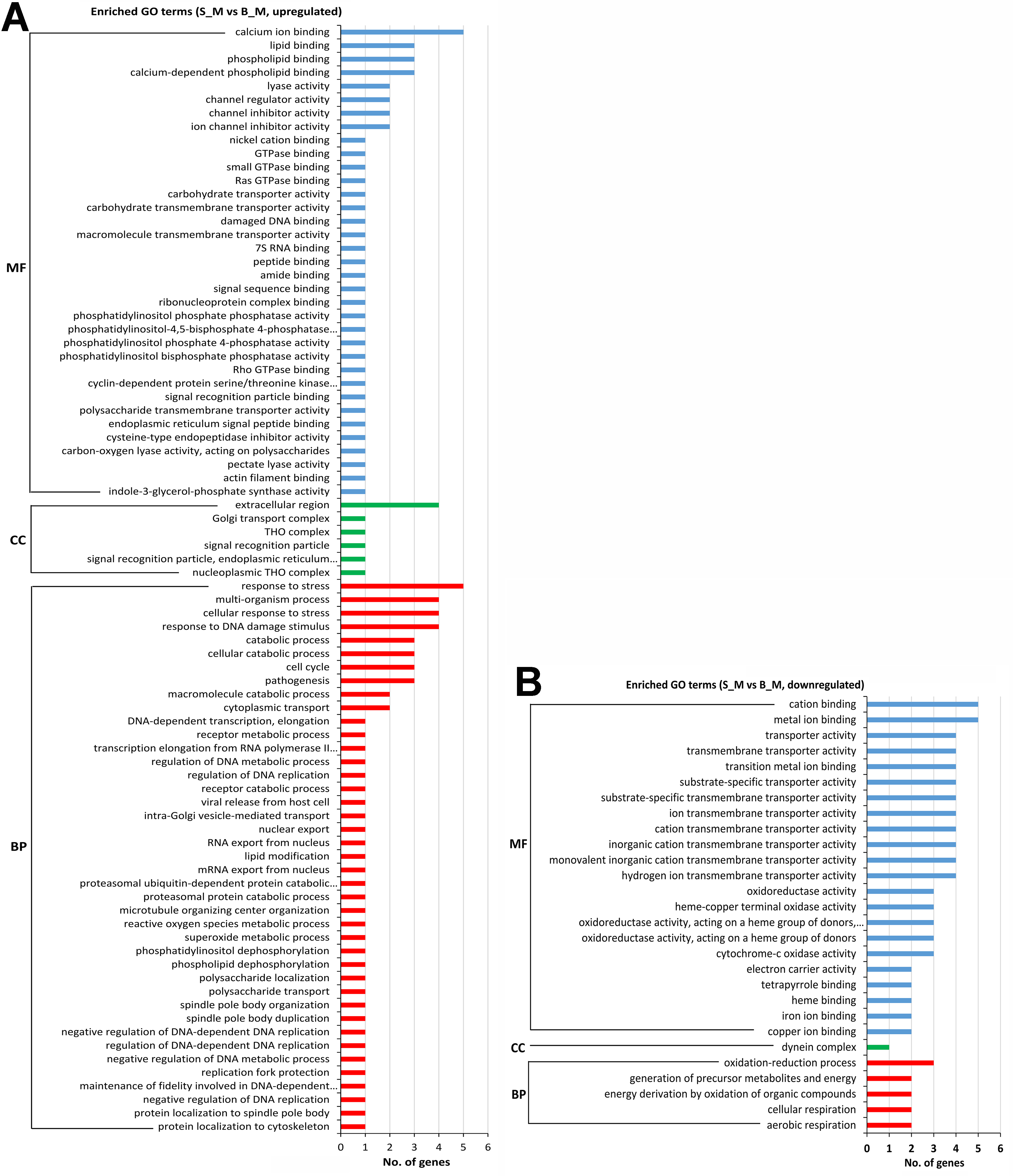

Supplement: FIGURE S1 — Sample relationships revealed by transcriptome profiles between worm samples. (A–F) Scatter plots of Pearson’s correlation analysis based on the transcriptome profiles between worm samples. The figures in the matrix R2 are the square of the correlation coefficient (r) between two samples. [file Data_Sheet_1.zip › Data Sheet 1/Figure S4.tif]

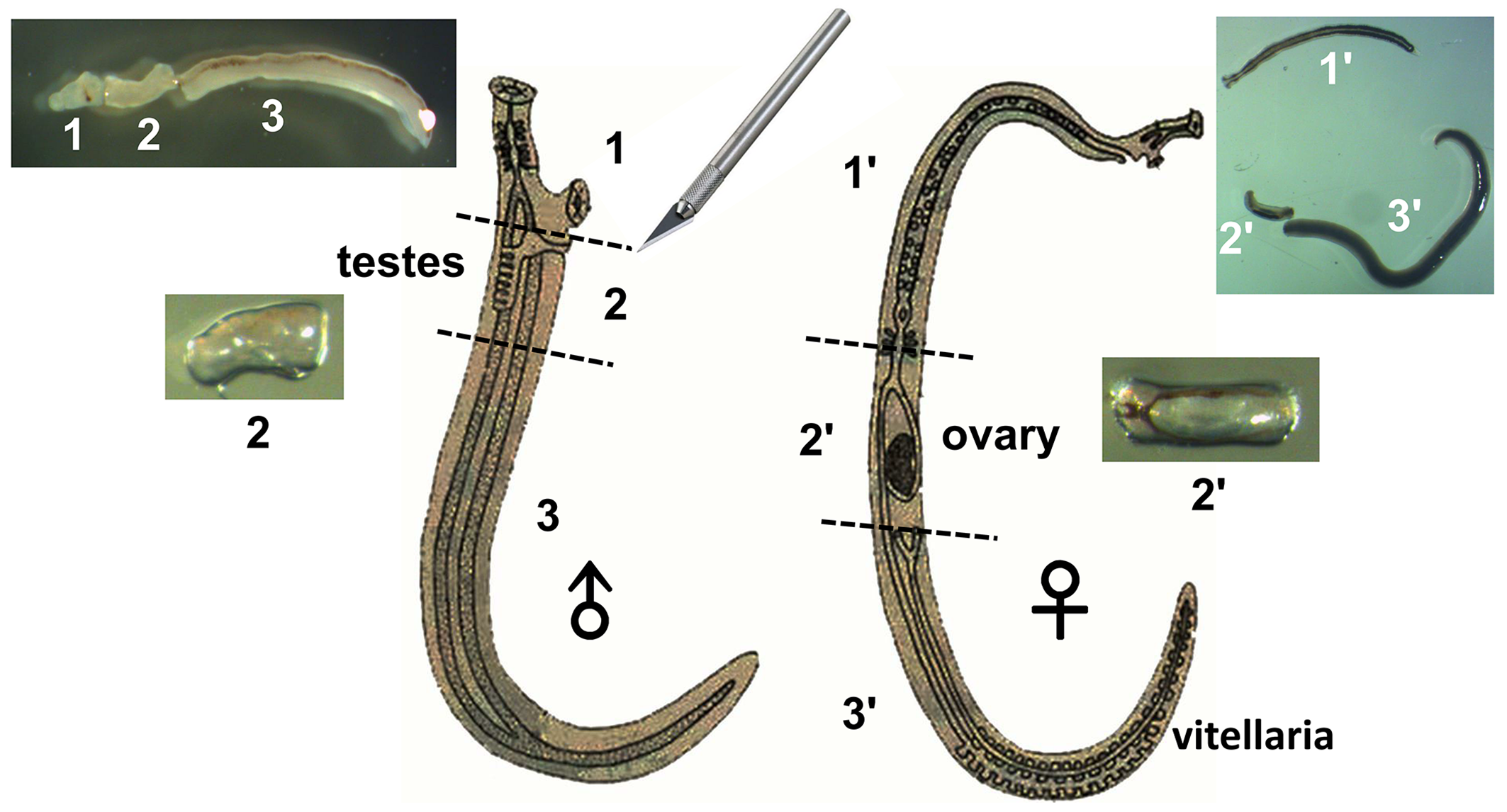

Supplement: FIGURE S1 — Sample relationships revealed by transcriptome profiles between worm samples. (A–F) Scatter plots of Pearson’s correlation analysis based on the transcriptome profiles between worm samples. The figures in the matrix R2 are the square of the correlation coefficient (r) between two samples. [file Data_Sheet_1.zip › Data Sheet 1/Figure S5.tif]

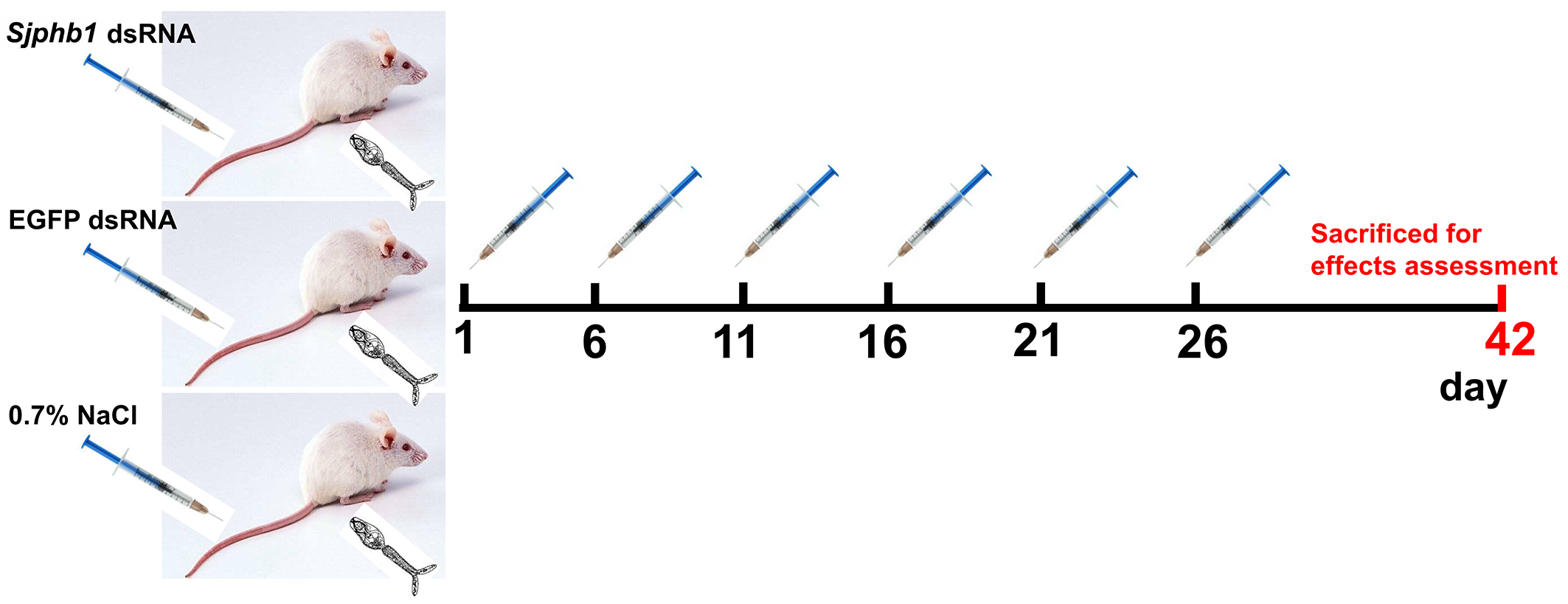

Supplement: FIGURE S1 — Sample relationships revealed by transcriptome profiles between worm samples. (A–F) Scatter plots of Pearson’s correlation analysis based on the transcriptome profiles between worm samples. The figures in the matrix R2 are the square of the correlation coefficient (r) between two samples. [file Data_Sheet_1.zip › Data Sheet 1/Figure S6.tif]
